# Synthesis of the large pore aluminophosphate STA-1 and its application as a catalyst for the Beckmann rearrangement of cyclohexanone oxime†

**DOI:** 10.1039/d4ta01132e

**Published:** 2024-05-13

**Authors:** Nuria González-Camuñas, Ángel Cantín, Daniel M. Dawson, Magdalena M. Lozinska, Joaquín Martínez-Triguero, James Mattock, Paul A. Cox, Sharon E. Ashbrook, Paul A. Wright, Fernando Rey

**Affiliations:** a Instituto de Tecnología Química (ITQ), Universitat Politècnica de València (UPV) – Consejo Superior de Investigaciones Científicas (CSIC) 46022 Valencia Spain frey@itq.upv.es; b Instituto de Tecnología Cerámica, Universidad Jaume I Campus Universitario Riu Sec, Avda. Vicente Sos Baynat s/n 12006 Castellón Spain; c EaStCHEM School of Chemistry, University of St Andrews Purdie Building, St Andrews KY16 9ST UK paw2@st-andrews.ac.uk; d School of Pharmacy and Biomedical Sciences, University of Portsmouth Portsmouth PO1 2DT UK

## Abstract

The preparation of stable large pore aluminophosphate (AlPO) zeotypes offers materials for applications in adsorption and catalysis. Here we report the synthesis of the pure AlPO with the SAO topology type (AlPO STA-1) using *N*,*N*′-diethylbicyclo[2.2.2]oct-7-ene-2,3:5,6-dipyrrolidine (DEBOP) as the organic structure directing agent in the presence of fluoride. The AlPO STA-1 can be rendered microporous (pore volume 0.36 cm^3^ g^−1^) *via* calcination and the calcined form remains stable in the presence of moisture. The structure of the dehydrated form has been established by Rietveld refinement (tetragonal *P*4̄*n*2, *a* = 13.74317(10) Å, *c* = 21.8131(5) Å, *V* = 4119.94(16) Å^3^). Multinuclear ^27^Al and ^31^P MAS NMR, together with 2D COSY and CASTEP NMR calculations, enables resolution and assignment of the signals from all crystallographically distinct Al and P framework sites. Structural elucidation of the as-prepared aluminophosphate-fluoride is more challenging, because of the presence of partially protonated OSDA molecules in the 3D-connected channel system and in particular because the fluoride ions coordinate with positional disorder to some of the Al atoms to give 5-fold as well as tetrahedrally-coordinated framework Al species. These are postulated to occupy Al–F–Al bridging sites, where they are responsible for distortion of the framework [*P*4̄*n*2, *a* = 13.3148(9) Å, *c* = 22.0655(20) Å, *V* = 3911.9(7) Å^3^]. Calcination and removal of fluoride ions and OSDAs allows the framework to expand to its relaxed configuration. The SAO topology type aluminophosphate can also be synthesised with small amounts of Si and Ge in the framework, and these SAPO and GeAPO STA-1 materials are also stable to template removal. IR spectroscopy with CO as a probe at 123 K indicates all have weak-to-mild acidity, increasing in the order AlPO < GeAPO < SAPO. These STA-1 materials have been investigated for their activity in the Beckmann rearrangement of cyclohexanone oxime to ε-caprolactam at 598 K: while all are active, the AlPO form is favoured due to its high selectivity and slow deactivation, both of which are a consequence of its very weak acid strength, which is nevertheless sufficient to catalyse the reaction.

## Abstract 2

Microporous AlPO_4_ STA-1 has been synthesised and studied by NMR crystallography; it is an effective catalyst for the Beckmann rearrangement of cyclohexanone oxime.

## Graphical abstract

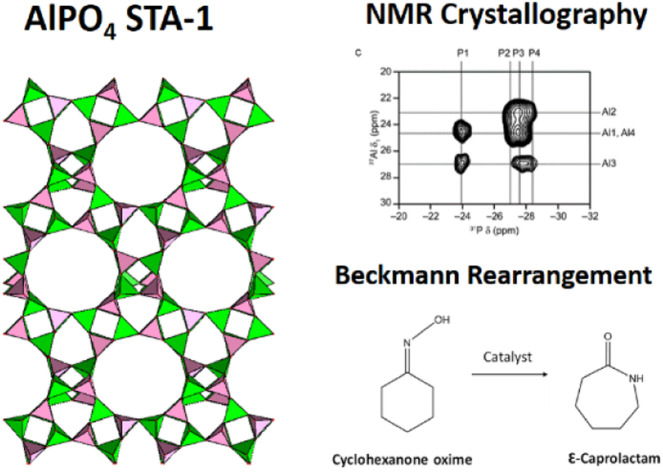

## Introduction

Microporous aluminophosphates (AlPOs) have been an important zeotype family since initial work in the early 1960s by D'Yvoire^1^ and breakthroughs in 1982 by Wilson *et al.*^2,3^ AlPO_4_ frameworks possess strict alternation of AlO_4_^−^ and PO_4_^+^ tetrahedra, giving electrically neutral materials, but their frameworks can be made anionic by substitution at framework cation sites to give metalloaluminophosphates (MAPOs) or silicoaluminophosphates (SAPOs).^4^ Up to now, 70 different structure types of aluminophosphate-based molecular sieves (AlPO_4_) have been reported, many of them displaying microporosity.

Aliovalent substitution of Al by a divalent cation such as Mg^2+^or Zn^2+^ or P^5+^ by Si^4+^^5−7^ leads to ion exchange capacity or solid acidity, where charge balance is established by protons, compensating organic or inorganic cations or defective Lewis acid sites. Small pore AlPO-based catalysts have found application in chemical and petrochemical processes such as the methanol-to-olefin conversion (MTO)^8–11^ and the selective catalytic reduction of NO using ammonia as reducing agent (NO-SCR)^12^ while large pore SAPOs have been investigated as catalysts for fine chemicals syntheses.^13^ There has also been considerable interest in other potential applications of aluminophosphates relying on the presence of structural porosity, which allows their use as molecular sieves in adsorption and separation processes.^14–16^

For most of these applications as adsorbents or catalysts, high adsorption capacity and fast diffusion are desirable properties. According to this, the aluminophosphate STA-1 (SAO framework type^17^) is of potential interest, because it has one of the lowest framework densities reported [13.4 tetrahedra per 1000 Å – among AlPOs, only AlPO-50 (AFY) and AlPO-37 (FAU) are less dense] and a pore space of intersecting 12-ring channels that has three-dimensional connectivity, which is rare among pure AlPO_4_ polymorphs. The AlPO STA-28 is one other recent example of a material with 3D connected 12-ring channels.^18^ STA-1 (SAO) has been synthesized previously as Mg- and Zn-containing AlPO_4_ materials,^19,20^ using diquinuclidine- and benzylpyrrolidine-based molecules [*e.g.*, for the latter, *S*-(−)-*N*-benzylpyrrolidine-2-methanol or benzylpyrrolidine] as organic structure directing agents, but not as AlPO_4_ or SAPO analogues. Previous studies showed that Mg- and Zn-containing AlPO_4_ materials with the SAO structure were unstable during calcination,^19,20^ which hindered their application as potential heterogeneous solid catalysts. Here, we describe the crystallization of STA-1 (SAO) as a pure aluminophosphate (AlPO_4_) using a previously unreported organic structure directing agent (OSDA) in the presence of fluoride. The ordering of Al and P in the framework and its high crystallinity renders the material amenable to characterisation by multinuclear NMR spectroscopy and X-ray crystallography. Full NMR signal assignment is achieved for the calcined, microporous form, while NMR analysis of the as-prepared AlPO provides an enhanced understanding of the structural role of fluoride in its synthesis. Additionally, it is possible to prepare AlPO STA-1 doped with low levels of Si and Ge and these are also stable to removal of the OSDA by calcination, giving highly porous 3D-connected large pore materials.

Microporous solids with weak acidity are known to be effective catalysts for the Beckmann rearrangement of cyclohexanone oxime to caprolactam, an important intermediate in the synthesis of Nylon-6.^21–26^ The industrial process uses oleum sulphuric acid as catalyst for this reaction and so has important drawbacks from pipeline corrosion, safety and environmental aspects.^27^ Consequently, the substitution of the homogeneous sulphuric acid catalyst by solid acid catalysts has been widely discussed in articles and patents.^13,28–30^ Among them, zeolites have been of major interest, and large and medium pore zeolites such as zeolite beta, faujasite or ZSM-5 have been investigated for this process.^31,32^ However, the strong acidity of such materials results in fast catalyst deactivation. Indeed, pure silica zeotypes such as silicalite (MFI) have been found to be good catalysts for this reaction because they possess only weakly acidic silanol groups.^33,34^ Aluminophosphate zeotypes have been described as a promising alternative, since they have a much milder acid strength than aluminosilicate zeolites, and SAPO-11 and SAPO-37 have been found to be active and selective for the rearrangement of cyclohexanone oxime to caprolactam. The unidirectional 10R pore topology of SAPO-11 strongly restricts its applicability due to diffusional limitations of reactant and products whereas the 3D large pore SAPO-37 is more suitable.^13^ Furthermore, the introduction of mesoporosity into SAPO-37 has been shown to greatly extend its catalytic lifetime.^13^ However, the lack of hydrolytic stability of SAPO-37 in the calcined form is a drawback for its large scale application as a catalyst.^35^

Here we report the catalytic performance of the AlPO form of STA-1 in the gas phase Beckmann rearrangement and compare its performance with that of STA-1 doped with Si and Ge and with weakly acidic SAPO-37, another 3D connected large pore aluminophosphate, a modification of which has recently been reported as an efficient catalysis for this reaction.^13^

## Methodology

**Scheme 1:**
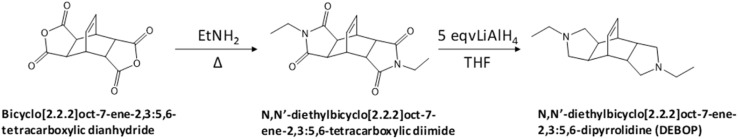
Synthesis of organic structure directing agent *N*,*N*′-diethylbicyclo[2.2.2]oct-7-ene-2,3:5,6-dipyrrolidine (DEBOP).

*N*,*N*′-diethylbicyclo[2.2.2]oct-7-ene-2,3:5,6-dipyrrolidine (DEBOP) was synthesized in two steps (Scheme 1) from bicyclo[2.2.2]oct-7-ene-2,3:5,6-tetracarboxylic dianhydride according to the reported procedure.^36^

For synthesis of the diimide, 81 mmol of commercially available bicyclo[2.2.2]oct-7-ene-2,3:5,6-tetracarboxylic dianhydride (Sigma-Aldrich) were heated at 348 K for 84 h with 200 mL of an ethylamine water solution (70 wt%, Sigma-Aldrich). Then, the mixture was partially concentrated under vacuum and the precipitated solid was filtered and vacuum dried, providing the diimide in quantitative yield.

For the reduction of diimide, 244 mmol of LiAlH_4_ were suspended in 300 mL of anhydrous THF under a N_2_ atmosphere. Following, 49 mmol of the diimide were added in small portions to the LiAlH_4_ suspension placed at 273 K (ice bath temperature). The mixture was heated under reflux for 6 h, and then stirred at room temperature overnight. Finally, the reaction was quenched by addition of distilled H_2_O (10 mL) and 15% aqueous solution of NaOH (10 mL). After 30 min stirring at room temperature the solution was filtered and the solvent partially evaporated. The paste was mixed with water and the amine extracted with CH_2_Cl_2_. The organic extract was dried on Na_2_SO_4_ and concentrated to dryness providing the desired DEBOP diamine (yield = 87%).

The aluminophosphate was prepared under hydrothermal conditions by using DEBOP as OSDA. DEBOP and orthophosphoric acid (85 wt%, Sigma-Aldrich) were dissolved in deionized water; next, aluminium isopropoxide (99 wt%, Sigma-Aldrich) was added. The mixture was stirred for 2 h until a homogeneous gel was achieved. Then hydrofluoric acid (50 wt%, Sigma-Aldrich) was added until the pH was adjusted to 7 and the synthesis gel (overall composition Al_2_O_3_: 1.3 P_2_O_5_: 1.6 DEBOP: 1.1 HF: 180 H_2_O) was stirred for a further 1 h. All handling of HF was conducted within a fume hood, wearing protective glasses, lab coat and gloves specifically designed for HF (certified under ISO 374:1-2016 Type A AKLOPS). This gel was sealed in a Teflon-lined autoclave and heated without stirring under autogenous pressure at 448 K for 24 h (Table 1). The resulting solid was isolated by filtration, washed with distilled hot water and dried at 373 K overnight. To remove the OSDA, the solid was calcined at 923 K for 18 h in air.

**Table 1:** Synthesis conditions for the different aluminophosphate forms of STA-1

Samples	Gel composition	Temp.(K)	Time (h)
AlPO-SAO	Al_2_O_3_: 1.3 P_2_O_5_: 1.6 DEBOP: 1.1 HF: 180 H_2_O	448	24
SAPO-SAO	1.2 P_2_O_5_: 0.2 SiO_2_: Al_2_O_3_: 1.6 DEBOP: 0.8 HF: 180 H_2_O	463	72
GeAPO-SAO	1.3 P_2_O_5_: 0.2 GeO_2_: 0.9 Al_2_O_3_: 1.6 DEBOP: 180 H_2_O	448	24

The synthesis gel compositions and synthesis conditions of the silicoaluminophosphate (SAPO STA-1) and the germanium–aluminophosphate (GeAPO STA-1) are described in Table 1. Fumed silica and germanium oxide were used as sources of silicon and germanium, respectively. The silicon or germanium sources were introduced just after addition of aluminium isopropoxide into the gel. While HF was added in the SAPO synthesis, where, as in the AlPO synthesis, it was required to give STA-1 product, none was required in the GeAPO preparation to achieve crystalline STA-1. In each case a minor amount of pure AlPO-SAO was introduced into the synthesis gels as seeds (accounting for 5 wt% of the total P and Al content in the final synthesis gel compositions).

The morphology of the samples was measured by field emission scanning electron microscopy (FESEM) on a Zeiss Ultra-55 electron microscope equipped with an energy dispersive X-ray detector (EDS). Thermogravimetry of all samples was performed using two different instruments, Mettler Toledo TGA/SDTA851e and Netzsch STA449 F3 Jupiter at a heating rate of 10 K min^−1^ up to 1073 K in flowing air. N_2_ adsorption isotherms were measured volumetrically at 77 K using Micromeritics ASAP 2420 and Micromeritics TriStar 300 on previously calcined samples. The samples were first degassed for 24 h at 673 K and high vacuum, in order to remove any adsorbed molecules. For ICP-AES analysis, the samples were digested in a mixture of concentrated aqueous acids (HNO_3_ : HF : HCl in a volumetric ratio of 1 : 1 : 3 and thus diluted to a volumetric ratio 1 : 10 in distilled water). The measurements were carried out in a Varian 715 ES spectrophotometer using commercial standards of each of the elements to make the corresponding calibration curves. Using this technique, the content of aluminium, phosphorus, silicon and other metals in the solids obtained was quantified and compared. Elemental analysis (CHN) measurements were carried out on a Eurovector EuroEA3000 using sulfanilamide as a reference.

The crystallinity of as prepared and calcined samples was confirmed by laboratory powder X-ray diffraction (PXRD) using PANalytical CUBIX diffractometer equipped with an X'Celerator detector, using Cu K_α_ X-rays (*λ*_1_ = 1.5406 Å, *λ*_2_ = 1.544 Å, *I*_2_/*I*_1_ = 0.5). The operating conditions of the equipment were 45 kV and 40 mA, with the measurement range (2*θ*) 2.0–40.0° and a step size of 0.020°. In order to investigate the structure of calcined and dehydrated AlPO STA-1 (SAO), the powder was loaded into 0.7 mm quartz glass capillary and dehydrated at 623 K at 1 × 10^−5^ mbar on a glass line for 12 h. The powder diffraction pattern of the sample was measured in Debye–Scherrer geometry on a Stoe STAD i/p diffractometer with monochromated Cu K_α1_ X-rays (*λ* = 1.54056 Å). The structure of the calcined and dehydrated AlPO STA-1 (SAO) sample was determined by Rietveld refinement against the XRD data, using the GSAS suite of programs.^37^ Starting framework models were adapted from a literature example with the unit cell modified to that derived from the diffraction pattern. The instrumental background was fitted automatically by using a Chebyshev function. The peak profiles were modelled using a Pseudo-Voigt function (type 2). The framework Al–O, P–O, O–O(Al), and O–O(P) distances were soft constrained to 1.72 Å (*σ* = 0.020 Å), 1.50 Å (*σ* = 0.020 Å), 2.82 Å (*σ* = 0.005 Å), and 2.50 Å (*σ* = 0.005 Å), respectively. For the as-prepared, dehydrated AlPO(F) STA-1, diffraction data was collected in the same way, except the dehydration was at 473 K. A starting model for the structure was obtained by including the OSDA in modelled positions of the structure of the calcined form and allowing the framework positions to refine, with the same constraints as above. Details of attempts to refine fluoride positions are described in the Results section. Cif files CSD 2333657 (calcined) and 2333658 (as-prepared) have been deposited *via* the CCDC/FIZ Karlsruhe deposition service.

Solid-state NMR spectra were recorded using Bruker Avance III spectrometers equipped with 9.4 and 14.1 T wide-bore superconducting magnets, at the Scottish High-Field NMR facility using a Bruker Avance Neo console equipped with a 18.8 T standard-bore superconducting magnet, or at the UK High-Field Solid-State NMR Facility using a Bruker Avance Neo console equipped with a 20.0 T wide-bore superconducting magnet. Samples were packed into standard Bruker magic angle spinning (MAS) zirconia rotors with outer diameters of 4, 3.2, 2.5 or 1.9 mm and rotated at the magic angle at rates of up to 25 kHz.

The ^13^C MAS NMR spectrum was recorded at 9.4 T using cross polarisation (CP) from ^1^H. A contact pulse (ramped for ^1^H) of 0.5 ms was applied with high-power (*ν*_1_ ≈ 100 kHz) TPPM-15 decoupling applied during acquisition. Signal averaging was carried out for 1024 transients with a recycle interval of 5 s. The ^19^F MAS NMR spectrum was recorded at 14.1 T with signal averaging for 16 transients with a recycle interval of 300 s. The ^29^Si MAS NMR spectrum was recorded at 9.4 T with signal averaging for 1128 transients with a recycle interval of 120 s. ^27^Al MAS NMR spectra were recorded at 9.4, 14.1, 18.8 and 20.0 T with signal averaging for between 256 and 2048 transients with a recycle interval of 0.5 or 1 s for the as-made materials and signal averaging for 128 transients with a recycle interval of 1 s for the calcined material (9.4 T only). A short pulse of flip angle ∼10–20° was used to ensure that spectra were as quantitative as possible. The multiple-quantum (MQ) MAS experiment was recorded at 9.4 T using an amplitude-modulated z-filtered experiment with signal averaging for 24 transients with a recycle interval of 0.5 s for each of 160 *t*_1_ increments of 71.43 μs. The spectrum was sheared and then scaled and referenced in the indirect dimension according to Pike *et al.*^38^

^31^P MAS NMR spectra were recorded at 9.4 T with signal averaging for between 10 and 32 transients with a recycle interval of 10 s (calcined AlPO) or 60 or 120 s (as-made materials). The ^31^P MAS NMR spectrum of the as-made AlPO was also recorded at 14.1 T with signal averaging for 144 transients with a recycle interval of 300 s to ensure complete relaxation. The ^27^Al–^31^P MQ-INEPT correlation experiments were recorded at 9.4 and 14.1 T with signal averaging for 336 transients with a recycle interval of 0.75 s for each of 254 *t*_1_ increments of 71.43 μs (9.4 T) and signal averaging for 288 transients with a recycle interval of 0.75 s for each of 180 *t*_1_ increments of 40 μs (14.1 T). The ^27^Al isotropic dimension was scaled and referenced according to Pike *et al.*^38^ The mixing time was 2.29 ms (=32 rotor periods) at 9.4 T and 2.24 ms (=56 rotor periods) at 14.1 T. Chemical shifts are reported in ppm relative to tetramethyl silane (^1^H and ^13^C), CFCl_3_ (^19^F), 1.1 M Al(NO_3_)_3_ in D_2_O (^27^Al) and 85% H_3_PO_4_ (^31^P) using secondary solid references of l-alanine (NH_3_ = 8.5 ppm, CH_3_ = 20.5 ppm), PTFE (CF_2_ = −122.7 ppm), Al(acac)_3_ (*δ*_iso_ = 0.0 ppm) and BPO_4_ (−29.6 ppm).

Geometry optimisation and calculation of NMR parameters were carried out using the CASTEP density functional theory (DFT) code^39^ employing the GIPAW algorithm,^40^ to reconstruct the all-electron wave function in the presence of a magnetic field. The initial structure was taken from the literature.^19^ Calculations used the GGA PBE functional, with core-valence interactions described by ultrasoft pseudopotentials,^41^ which were generated on the fly, accounting for scalar relativistic effects by using ZORA.^42^ A planewave energy cut-off of 60 Ry was used, and integrals over the Brillouin zone were performed using a Monkhorst–Pack grid with *k*-point spacing of 0.04 2π Å^−1^. Dispersive interactions were reintroduced using the scheme of Tkatchenko and Scheffler (TS)^43^ as implemented by McNellis *et al.*^44^ The isotropic shielding is given by *σ*_iso_ = (1/3) Tr{*σ*}, and the isotropic chemical shift, *δ*_iso_, by −(*σ*_iso_ − *σ*_ref_)/*m*, where *σ*_ref_ is a reference shielding and *m* is a scaling factor (ideally *m* = 1). Values used here were *σ*_ref_ = 564.0 ppm and *m* = 1 for ^27^Al and *σ*_ref_ = 283.38 and *m* = 1.28 for ^31^P. The magnitude of the quadrupolar coupling constant is given by *C*_Q_ = *eQV*_*ZZ*_/*h*, where *Q* is the nuclear quadrupole moment (for which a value of 146.6 mb was used for ^27^Al).^45^ The asymmetry parameter is given by *η*_Q_ = (*V*_*XX*_ − *V*_*YY*_)/*V*_*ZZ*_. The peak position in the isotropic dimension of the ^27^Al MQMAS spectrum is *δ*_1_ = (17/31)*δ*_iso_ + (32/93)*δ*_Q_, where *δ*_Q_ = (3000*P*_Q_/20*ν*_0_)^2^ and *P*_Q_ = *C*_Q_(1 + (*η*_Q_^2^/3))^1/2^.^38^

The position of OSDAs in the pores of STA-1 was modelled *via* a simulated annealing of a varying number of OSDA molecules followed by a periodic geometry optimisation performed using the Forcite module within the Materials Studio 2019 program.^46^ The COMPASSII forcefield was used with charges being calculated *via* charge equilibration (*Q*_Eq_).^47^ The smart algorithm was used to achieve optimisation. An atom-based summation method was used for both the electrostatic and van der Waals energy contributions.

Initially, the lowest energy configuration of the DEBOP OSDA was determined outside of the zeotype framework (see Fig. S1† for the configuration) and this was used for subsequent modelling, with a unit cell assigned *P*1 symmetry. Increasing numbers of molecules, up to the experimentally determined value of four per unit cell, were added and energy-minimised. The binding energies of varying numbers of OSDA moieties within an AlPO framework are presented in Table S1.† Computational Modelling approaches of this type have been used widely to identify the location of OSDA molecules inside host frameworks.^48–50^

Fourier Transform Infrared spectroscopy using CO as a probe molecule (FTIR-CO) was performed on a Nicolet 6700 FTIR instrument. First, a self-supported pellet of the material with a diameter of 20 mm and a weight of 15 mg was prepared and pre-treated in synthetic air at 673 K for 2 hours. The pellet was then transferred to a low-temperature cell, where it underwent a further pre-treatment in synthetic air at 473 K for 30 min, after which it was cooled to 123 K and a CO mixture (5% by volume in He) was supplied until saturation was achieved. Before the measurement, it was necessary to remove the physisorbed CO by evacuation under high vacuum. Finally, CO was desorbed by applying high vacuum as the temperature was slowly raised.

The Beckmann rearrangement was carried out in an isothermal fixed-bed tubular reactor with a diameter of 4 mm. The sample, sieved to yield a particle size of 0.2–0.4 mm, was introduced between two beds of silicon carbide (SiC, Merck). In the lower bed, 500 mg of SiC was introduced while 1.8 g was placed in the upper bed, which acts as a feed preheater. The catalysts used in this reaction were calcined *in situ* in the reactor, so the amount of catalyst was calculated taking into account the weight loss by thermogravimetric analysis (TGA) so that the weight of the calcined catalyst was 200 mg. The as-prepared materials were calcined for 18 h with an air flow of 80 mL min^−1^ at 923 K. Once calcined, the conditions were changed to 673 K and N_2_ flow of 50 mL min^−1^ for one hour and finally, reaction conditions were set, lowering the temperature to 598 K with an N_2_ flow of 33.3 mL min^−1^ and starting to feed by means of a peristaltic pump using 10 g L^−1^ solution of cyclohexanone oxime in ethanol at a weight hourly space velocity (WHSV) of 0.79 g_reactant_ g_cat_^−1^ h^−1^. To avoid product condensation, the reactor outlet was heated to 423 K. The reaction products were collected after 1 h in a collection trap in which a known amount of ethanol was previously added. After one hour, the collecting trap was changed and the sample for analysis in a gas chromatograph was prepared. For this purpose, the sample was collected in a vial with a known amount of internal standard (10 g mL^−1^ of a solution of mesitylene in ethanol) and subsequently analysed in a Varian 3900 gas chromatograph equipped with a HP5-25 capillary column. The injection volume was set at 1 μL, the injector temperature being 553 K and the FID detector temperature at 553 K. N_2_ was used as carrier gas, with a constant flow rate of 1 mL min^−1^. The temperature programme used for sample analysis was: 373 K, 1 min; 20 K min^−1^ up to 553 K, 25 min at 553 K.

## Results and discusssion

**Fig. 1:**
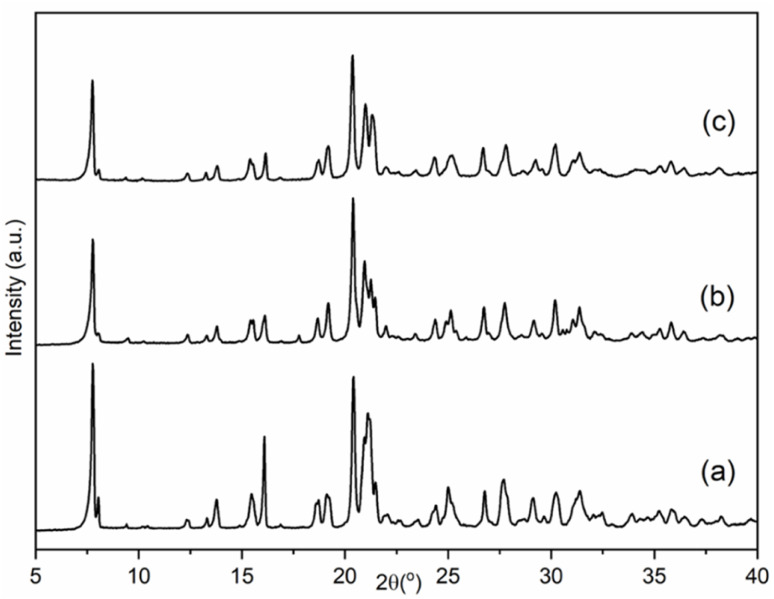
PXRD patterns of the as-made samples of (a) AlPO, (b) SAPO and (c) GeAPO forms of STA-1.

Reaction of aluminophosphate and doped aluminophosphate gels in the presence of the DEBOP OSDA and fluoride ions gave highly crystalline powders (Fig. 1), comprising crystallites *ca*. 1 μm in dimensions (Fig. S2†). The crystalline aluminophosphate contained fluorine, with a F/Al ratio estimated by EDS to be 0.18. The P/Al ratio was estimated to be 1.07 by EDS and 0.93 by ICP-OES, within experimental error of the ideal 1 : 1 ratio. The presence of organic template was confirmed by elemental analysis (Table S2†), which gave a C/N ratio of 7.9(1) consistent with that of the DEBOP OSDA (8).

**Fig. 2:**
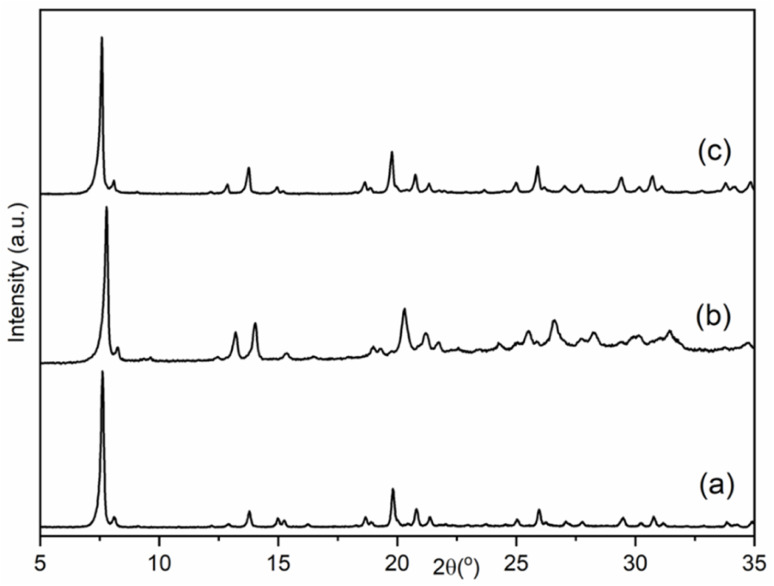
PXRD patterns of the calcined samples of (a) AlPO, (b) SAPO and (c) GeAPO forms of STA-1.

**Fig. 3:**
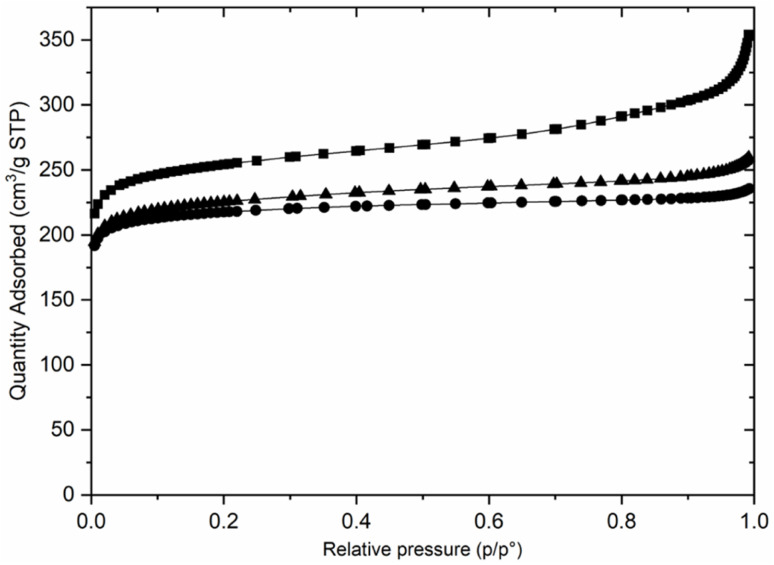
N_2_ adsorption isotherms at 77 K for calcined AlPO (■), SAPO (▲) and GeAPO (●) forms of STA-1.

Preliminary examination of the PXRD patterns of the AlPO indicated similarities to those reported for STA-1 materials, but also important differences, particularly in peak positions. Previous studies have shown that the crystal structures and particularly the unit cell dimensions of as-prepared AlPOs containing fluoride or hydroxide ions coordinated to framework aluminium as charge balancing species are often significantly different from undistorted AlPOs or framework substituted SAPOs or MAPOs. This arises from changes in framework geometry that result when fluoride or hydroxide ions are coordinated to framework Al, expanding its coordination above 4.^51,52^ In these cases, calcination of the solids resulted in removal of the bound fluoride or hydroxide anions and relaxation of the framework. Therefore, the as-prepared materials of this work were calcined in air at 923 K, conditions that TGA (Fig. S3†) indicated would result in the removal of all organic template and any fluoride or hydroxide present. PXRD of the calcined solids showed the materials were crystalline after calcination and subsequent exposure to air, and that they resembled those reported for STA-1 (Fig. 2).^19,20^ N_2_ adsorption of the calcined sample at 77 K gives Type I isotherms (Fig. 3) indicating the calcined solids are microporous with pore volumes of 0.36 cm^3^ g^−1^ for AlPO STA-1, 0.34 cm^3^ g^−1^ for SAPO STA-1 and 0.33 cm^3^ g^−1^ for GeAPO STA-1.

**Fig. 4:**
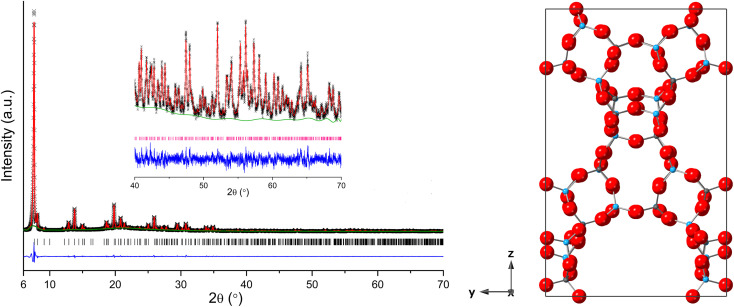
(Left) Rietveld plot of PXRD data (*λ* = 1.5406 Å, *T* = 298 K) of calcined and dehydrated AlPO STA-1 (Observed – black, calculated – red, difference – blue, phase – pink and background – green). (Right) Structure of calcined and dehydrated (*P*4̄*n*2) AlPO STA-1 obtained from Rietveld refinement (Al = blue spheres, P = grey spheres and O = red spheres).

The PXRD of the calcined AlPO STA-1 material was indexed as tetragonal, *a* = 13.74317(10) Å and *c* = 21.8131(5) Å, which was sufficiently similar to that reported for the undistorted as-prepared MgAPO STA-1 structure^19^ to allow Rietveld analysis of the calcined, dehydrated AlPO STA-1 using the published atomic coordinates in *P*4̄*n*2 as a starting model (this allows Al and P ordering). Refinement gave a good fit to the observed data (Fig. 4), with realistic Al–O and P–O bond lengths and O–Al–O and O–P–O bond angles (Tables S3–S5†).

As reported previously, the structure has 4 crystallographically-distinct Al sites and 4 distinct P sites. The unit cell formula is Al_28_P_28_O_112_, with full tetrahedral connectivity and the cation site labelling is such that the ratio of the multiplicities of the sites is Al_1_ : Al_2_ : Al_3_ : Al_4_ = P_1_ : P_2_ : P_3_ : P_4_ = 2 : 2 : 2 : 1, as described in Table 2 and Fig. S4.† The overall pore structure contains straight channels bounded by 12Rs along the *a* and *b* directions, with their centres at heights *ca. z* = 0.10 and 0.60 and *z* = 0.40 and 0.9 in the unit cell, respectively, which partially intersect to give three dimensional large pore connectivity, with a zigzag channel running parallel to the *c* direction.

**Table 2:** T-site nearest tetrahedral cation neighbour environments in SAO-type AlPO_4_

Site	Multiplicity	Neighbours	Site	Multiplicity	Neighbours
Al_1_	2	P_1_ P_2_ P_3_ P_3_	P_1_	2	Al_1_ Al_3_ Al_3_ Al_4_
Al_2_	2	P_2_ P_2_ P_3_ P_4_	P_2_	2	Al_1_ Al_2_ Al_2_ Al_4_
Al_3_	2	P_1_ P_1_ P_3_ P_4_	P_3_	2	Al_1_ Al_1_ Al_2_ Al_3_
Al_4_	1	P_1_ P_1_ P_2_ P_2_	P_4_	1	Al_2_ Al_2_ Al_3_ Al_3_

As a result of the strict Al, P ordering in framework cation sites and the well-defined and fully tetrahedrally-connected crystal structure, dehydrated calcined STA-1 gives readily interpretable ^27^Al and ^31^P MAS NMR spectra, when assisted by DFT calculations as described in the Methodology section.

**Fig. 5:**
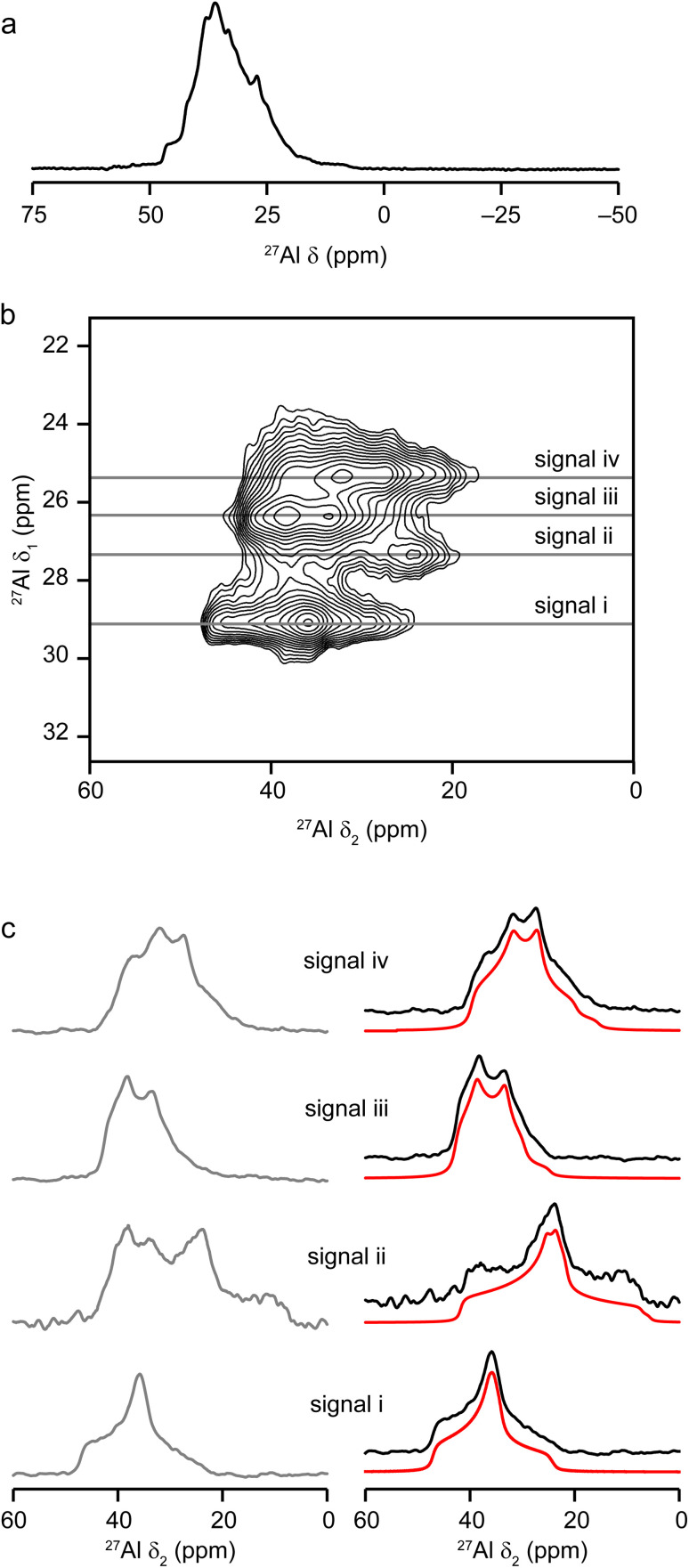
(a) ^27^Al (9.4 T, 14 kHz MAS) and (b) ^27^Al (9.4 T, 14 kHz MQ MAS) NMR spectra of calcined AlPO STA-1. (c) Lineshapes extracted from the ridges (grey) shown in (b) and lineshapes corrected for overlap of ridges (black) with fits to the experimental lineshapes (red) using the parameters reported in Table 3.

The ^27^Al MAS NMR spectrum, shown in Fig. 5a, contains a single broad peak corresponding to tetrahedral AlO_4_ species. However, a two-dimensional multiple-quantum (MQ) MAS NMR spectrum (Fig. 5b) shows four distinct ridges. By taking sections parallel to *δ*_2_, four lineshapes can be extracted (grey lines in Fig. 5c). Note that signal iii overlaps signals ii and iv in *δ*_1_ of the MQ MAS spectrum. As such, the contribution from signal iii was subtracted from the ridges extracted for signals ii and iv to reveal the true lineshapes arising from signals ii and iv (black lines, Fig. 5c), from which experimental NMR parameters can be extracted for all four signals using analytical fitting (red lines, Fig. 5c). Analytical fits are in red, with the parameters used to give these. The good agreement between the DFT-calculated NMR parameters from the energy minimised structure and those derived from the matched experimental lineshapes (Table 3) gives confidence in the ^27^Al NMR assignments.

**Table 3:** Experimental (exp) and calculated (calc) NMR parameters for calcined AlPO STA-1. Approximate errors on the experimental values are given in parentheses. In the ‘*δ*_iso_’ column, the experimental (exp) value given is that of the corresponding signal, labelled i–iv, shown in the ^27^Al MQ MAS NMR of Fig. 5

Site	*δ*_iso_ (ppm)		*δ*_1_ at 9.4 T (ppm)		[*C*_Q_] (MHz)		*η*_Q_	
	Exp	Calc	Exp	Calc	Exp	Calc	Exp	Calc
Al_1_	44(1) – iii	45.3	26.4(1)	27.2	3.67(4)	3.54	0.39(5)	0.44
Al_2_	40(1) – iv	42.2	25.3(1)	26.3	4.13(4)	4.08	0.59(5)	0.49
Al_3_	47(1) – i	49.2	29.1(1)	30.3	3.84(3)	3.86	0.93(5)	0.85
Al_4_	42(1) – ii	43.3	27.3(2)	27.9	4.81(2)	4.40	0.90(5)	0.80
P_1_	−23.9(2)	−23.99						
P_2_	−27.0(2)	−27.08						
P_3_	−27.6(2)	−28.05						
P_4_	−28.3(2)	−28.08						

**Fig. 6:**
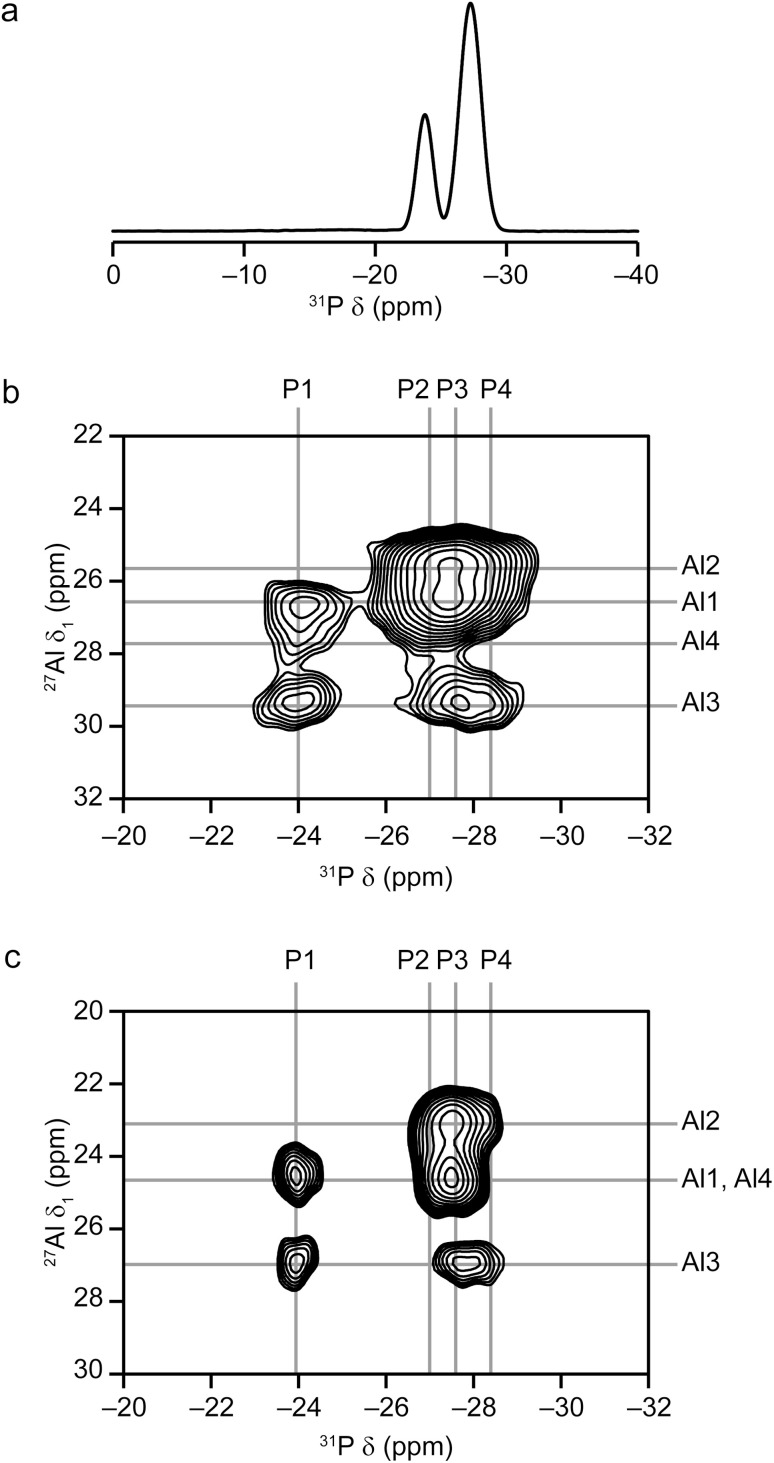
(a) ^31^P (9.4 T, 14 kHz MAS) NMR spectrum of calcined AlPO STA-1. (b and c) ^27^Al – ^31^P (14 kHz MAS) MQ-INEPT correlation spectra of calcined AlPO STA-1 recorded at (b) 9.4 T and (c) 14.1 T.

The ^31^P MAS NMR spectrum, shown in Fig. 6a, has two resolved peaks, at −23.9 and −27.5 ppm, with intensities in the ratio 2 : 5, indicating complete overlap of three of the resonances from the four crystallographic sites. The DFT calculations (summarised in Table 3) indicate that the peak at −23.9 ppm corresponds to P_1_, whereas the other three P sites are expected to overlap.

Additional information on the AlPO STA-1 structure, and in particular confirmation of the assignment of the NMR resonances, was obtained by ^27^Al–^31^P MQ-INEPT experiments (shown in Fig. 6b and c), which reveal pairs of spatially close Al and P. This allows full assignment of all signals when combined with information from the DFT calculations. One particular benefit of the two-dimensional MQ-INEPT spectrum is that the ^31^P signal at −27.5 ppm in Fig. 6a can now be seen to have three distinct contributions at −27.0, −27.6 and −28.3 ppm. Note that the ^27^Al *δ*_1_ is field dependent and the following discussion quotes values from the experiment at 9.4 T (Fig. 6b). Correlation of the P_1_ (−23.9 ppm) signal with three Al signals but not that at 25.3 ppm (*δ*_1_) indicates that to be Al_2_. Al_3_ is calculated to have the highest chemical shift, and so is likely to correspond to the signal at 29.1 ppm (in *δ*_1_). The Al_3_ signal correlates to all P signals apart from that at −27.0 ppm, suggesting its assignment as P_2_. The ^31^P signal at −28.3 ppm correlates only to the Al_2_ and Al_3_ signals, suggesting its assignment as P_4_ and confirming the assignments of these two Al signals (the correlation to only Al_2_ and Al_3_ is much more apparent in the spectrum recorded at 14.1 T (Fig. 6c) for a different sample of calcined AlPO STA-1). Therefore, the remaining ^31^P signal at −27.6 ppm corresponds to P_3_ and the ^27^Al signals at 26.4 and 27.1 ppm (in *δ*_1_) correspond to Al_1_ and Al_4_. The DFT calculations indicate that Al_1_ should have the smaller *C*_Q_ and *η*_Q_ (see Methods for definition) which would suggest an assignment of the signal at 26.4 ppm to Al_1_ and that at 27.2 ppm to Al_4_. This assignment is also in agreement with the observed spectral intensities (Table 3).

We then investigated the structure and chemistry of the as-prepared AlPO(F) in the light of information from the calcined STA-1. Le Bail refinement against the PXRD gave a good fit for a tetragonal unit cell, with *a* = 13.3148(9) Å and *c* = 22.0655(20) Å, similar to the calcined AlPO_4_ STA-1 but with anisotropic changes in the cell dimensions (*a*, *b* shorter by 0.43 Å, *c* longer by 0.25 Å) and a cell volume 5% lower than that of the calcined form (Table S3†).

**Fig. 7:**
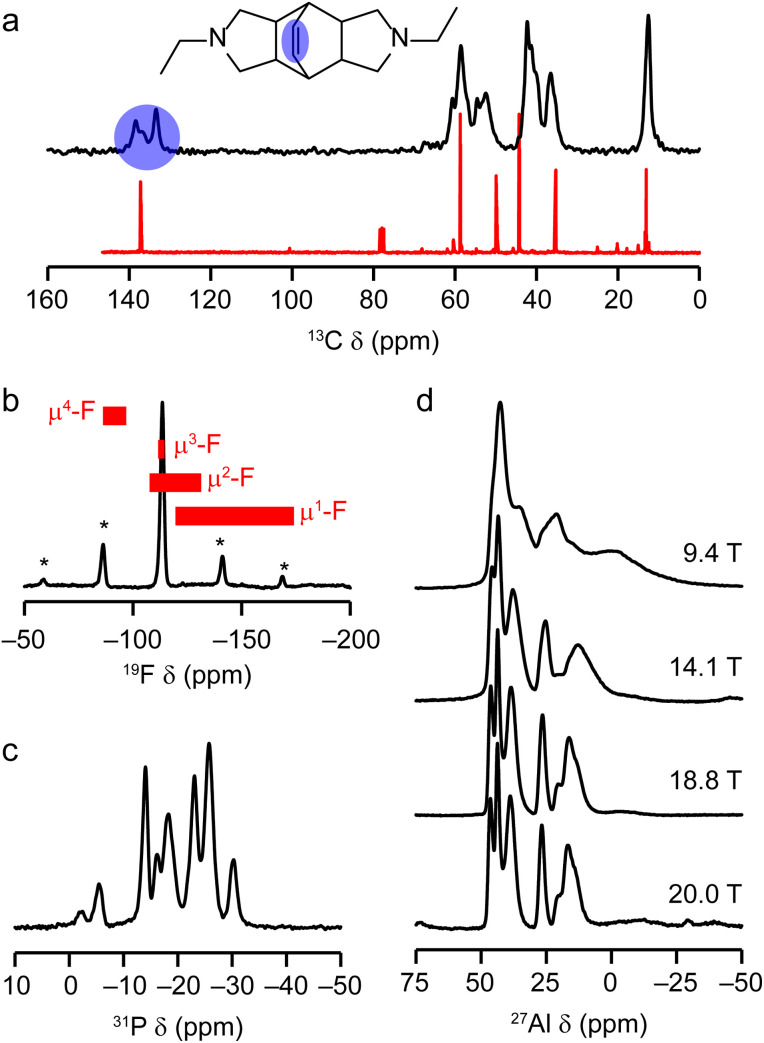
(a) ^13^C (9.4 T, 14 kHz CP MAS) NMR spectrum of as-prepared STA-1. The structure of the OSDA is shown in the inset and the alkene signals are highlighted in blue. For comparison, the ^13^C NMR spectrum of DEBOP in CDCl_3_ is shown in red. (b) ^19^F (14.1 T, 15.5 kHz MAS) NMR spectrum of as-prepared STA-1. Asterisks indicate spinning sidebands and the red boxes indicate literature ^19^F chemical shift ranges for μ^4^-F, μ^3^-F, μ^2^-F and μ^1^-F. (c) ^31^P (14.1 T, 14 kHz MAS) NMR spectrum of as-prepared STA-1. (d) Variable-field ^27^Al MAS NMR spectra of as-prepared STA-1 (9.4 T, 14 kHz MAS; 14.1 T, 14 kHz MAS; 18.8 T, 25 kHz MAS and 20.0 T, 12.5 kHz MAS).

Solid-state NMR spectroscopy was used to give some further insight into the structure of the as-prepared AlPO(F). Fig. 7 shows the ^13^C CP MAS and ^19^F, ^31^P and ^27^Al MAS NMR spectra of the material. Fig. 7a shows a comparison of the ^13^C CP MAS NMR spectrum of the DEBOP within the AlPO(F) framework and the solution-phase NMR spectrum of DEBOP in CDCl_3_. The DEBOP appears to remain intact within the AlPO, but the number of alkene signals (at 133.5, 136.8 and 138.6 ppm, highlighted in blue in Fig. 7a) suggests at least two different environments of the molecule. Elemental CHN analysis (Table S2†) is consistent with four intact molecules of DEBOP per unit cell within the pores of the AlPO(F) and fluorine analyses suggest that there are five F^−^ ions per unit cell, which would indicate that the amine molecules are either singly or doubly protonated to achieve charge balance.

The ^19^F MAS NMR spectrum in Fig. 7b contains a single broad resonance, at −113 ppm, indicating all F are in very similar environments. Comparison with the published ^19^F MAS NMR spectra of other fluorinated AlPO frameworks^53–61^ indicated by the shift ranges shown in red in Fig. 7b, suggests all fluoride ions occupy O_4_Al–F–AlO_4_ bridging sites. Consideration of the undistorted SAO structure indicates the shortest Al⋯Al distance where Al–F–Al bridging could occur is in the *sti* cage between Al(2) and Al(3). A similar position is located for F in the *sti* cages of UiO-7 (Fig. S5†).^53,62^

The ^31^P and ^27^Al MAS NMR spectra of the as-prepared AlPO(F), shown in Fig. 7c and d, are considerably more complex than those of the calcined material. The ^31^P MAS NMR spectrum shows at least eight signals of varying intensity and width, while the ^27^Al MAS NMR spectrum shows numerous resonances in chemical shift ranges consistent with both tetrahedral and 5-fold coordinate Al species (denoted Al^IV^ and Al^V^, respectively). As the second-order quadrupolar broadening of the ^27^Al signals is inversely proportional to B_0_, the use of higher magnetic field strengths (here 18.8 and 20.0 T) drastically improves the spectral resolution (as seen in Fig. 7d) and allows an Al^IV^ : Al^V^ ratio of around 16.5(3) : 11.5(3) in the unit cell to be determined (*i.e.*, slightly less than 3/7 of the Al are Al^V^). The results from these one-dimensional NMR spectra suggest that disorder within the OSDA and F locations result in considerable structural disorder in the overall crystal. Nevertheless, the elemental analysis and multinuclear NMR data is consistent with a model for the as-prepared AlPO(F) that has the framework connectivity of the fully tetrahedral AlPO_4_, but with DEBOP OSDAs within the pores and bridging F^−^ ions in a number of locations.

**Fig. 8:**
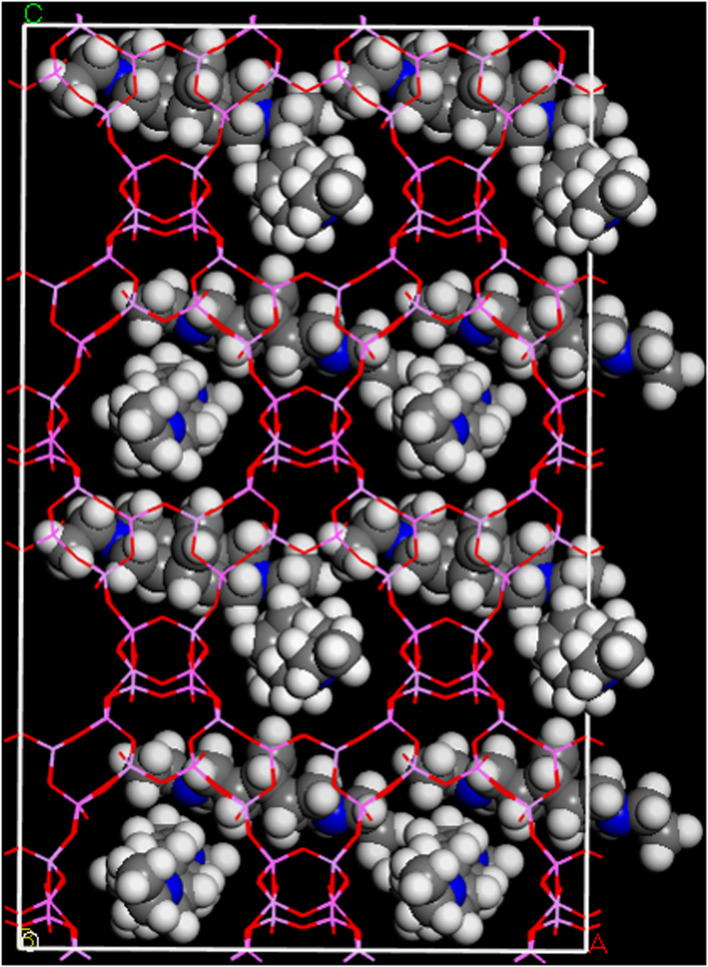
Positions of four OSDA moieties per unit cell within a 2 × 2 × 2 supercell of AlPO STA-1 (SAO) framework calculated *via* an adsorption locator calculation followed by a geometry optimisation within Forcite with the COMPASSII forcefield and *Q*_Eq_ charges (OSDA atoms, C – grey, N – blue, H – white).

Computer modelling was used to suggest starting positions for the four DEBOP molecules per unit cell, modelled as deprotonated species in an undistorted AlPO_4_ framework where the overall symmetry was reduced to *P*_1_. The filling of four sites in the unit cell were calculated to be favourable energetically, as given in Table S1.† Energy-minimised positions for the molecules were found with the long axes of the OSDAs running along the channels, with good filling of the available pore space, as shown in Fig. 8.

Including these species into the structural model and allowing distortion of the framework but retaining reasonable Al–O and P–O bond lengths (Table S6†), it was possible to achieve a reasonable fit to the PXRD data of the dehydrated, as-prepared AlPO(F) by Rietveld refinement (Fig. S6, Tables S3 and S7†). Attempts to locate the fluoride ions by difference Fourier analysis were unconvincing, but it was possible to refine some F occupancy in the Al_2_–F–Al_3_ bridging site identified by a consideration of the structure and the ^19^F MAS NMR chemical shift data. The final fit is satisfactory (Fig. S6†) and supports a model where the connectivity of the as-prepared AlPO_4_ framework is already present in the as-prepared form and retained upon calcination.

These results indicate that partially protonated DEBOP acts as a template for the AlPO (SAO) structure, and fluoride ions that occupy bridging positions between Al atoms charge balance the material. It is likely to be the presence of these fluoride ions that strongly distorts the framework, as seen in other aluminophosphates,^51,52^ with the consequence that the PXRD is markedly different from that observed previously for as-prepared STA-1 MAPO materials (M = Mg, Zn), where the charge balance is instead achieved by substitution of M^2+^ for Al^3+^.

Calcination of the as-prepared AlPO(F) removes the organic template molecules and fluoride ions completely, leaving the fully tetrahedrally-connected AlPO_4_ STA-1 with high crystallinity. This is the first time that this composition of SAO has been prepared. Nitrogen porosimetry of the calcined material (Fig. 3) shows that the high potential pore volume of the SAO framework has to a large extent been realised. The micropore volume of 0.36 cm^3^ g^−1^ compares favourably with other large pore zeolites and zeotypes. For example, zeolite Beta, which also possesses intersecting 12-ring channels running across *a* and *b* in a tetragonal structure, is reported to have a micropore volume of 0.22 cm^3^ g^−1^.^63^

Silicoaluminophosphate and germanium–aluminophosphate analogues of STA-1 were also synthesised, as indicated by the similarity of their PXRD patterns to that of as-prepared AlPO STA-1 (Fig. 1) as well as by elemental analyses of the microcrystalline powders (Table S2†), which indicate the Si or Ge contents are 1.8 and 2.3 atoms per unit cell (*i.e.* (Al + P)/M(IV) ≈ 25) in the SAPO-STA-1 and GeAPO-STA-1 samples, respectively. The similarity of the GeAPO to the AlPO–F pattern, even though no fluoride was employed in its synthesis, suggests that hydroxide ions bind to Al sites in a similar way to the fluoride ions, and result in a similar structural distortion. Bridging hydroxyl groups of this type have previously been observed in aluminophosphates.^64^

The ^31^P MAS NMR spectrum of SAPO STA-1 (black line, Fig. S7a†) is very similar to the spectrum of the pure AlPO (red line, Fig. S7a†), although the resonances are significantly broadened, indicative of local disorder. All resonances are observed in roughly the same relative intensities (when different linewidths are accounted for), such that no site-specific Si substitution is evident. The ^27^Al MAS NMR spectrum of SAPO STA-1 (black line, Fig. S7b†) shows the presence of Al^IV^ and Al^V^, with a profile that is similar to the pure AlPO (red line, Fig. 7b). However, the ^27^Al resonances for the SAPO are broader than for the AlPO, indicative of some local disorder (which would be expected in a doped framework). The ^29^Si MAS NMR spectrum of SAPO STA-1 is shown in Fig. S8† and gives a very weak signal even when recorded for 688 transients (compared with the 200–400 required to achieve high signal to noise for a standard aluminosilicate zeolite). This suggests that the sample does not contain much Si, but the peak maximum (at around −90 ppm) is indicative of Si substituting onto the P site (*i.e.*, Si(OAl)_4_ species).

The ^31^P MAS NMR spectrum of GeAPO STA-1 (black line, Fig. S9a†) is similar to the spectrum of the pure AlPO (red line, Fig. S9a†), although the resonances are slightly broadened, indicative of local disorder. In addition, some resonances have changed in intensity, and a new resonance is observed at 1.2 ppm, which was not present for the AlPO. However, further conclusions cannot be drawn since the GeAPO was prepared in the presence of OH^−^ rather than F^−^. The ^27^Al MAS NMR spectrum of as-prepared GeAPO STA-1 (black line, Fig. S9b†) shows the presence Al^IV^ and Al^V^, with a profile that is again similar to the pure AlPO (red line, Fig. S9b†), although the line broadening is again evident. Upon calcination, the SAPO and GeAPO retain crystallinity and microporosity and their PXRD patterns indicate they are isostructural with the AlPO. For the GeAPO, therefore, this is (to our knowledge) the first report of a Ge-containing 3D-connected large pore aluminophosphate, although some smaller pore GeAPOs have been reported.^65,66^

**Scheme 2:**
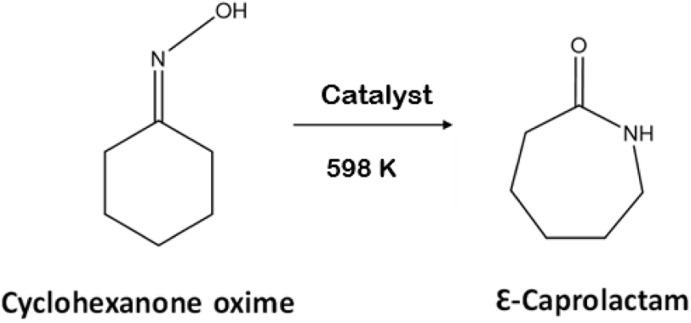
Beckmann rearrangement of cyclohexanone oxime.

AlPO-based materials typically exhibit mild acid properties, making them suitable catalysts for reactions with low acid demand, such as the Beckman rearrangement of cyclohexanone oxime (Scheme 2). All materials were therefore investigated as microporous catalysts for this reaction, with the composition expected to give a range of solid acid behaviour.

**Fig. 9:**
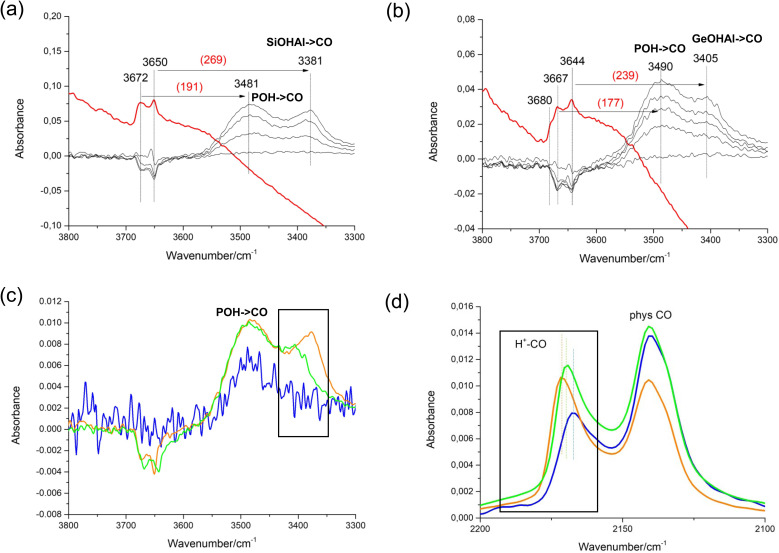
IR spectroscopy of the STA-1 materials before and after CO adsorption at 123 K on samples evacuated at 673 K. In (a) and (b) the spectra of dehydrated SAPO STA-1 and GeAPO STA-1 are compared with the difference spectra after CO has been dosed onto the solids. The difference spectra after dosing of CO on the AlPO, SAPO and GeAPO forms with CO are given (c) for the hydroxyl region and (d) the CO stretch (AlPO – blue line, GeAPO – green, SAPO – orange).

We characterised the acid properties of our STA-1 samples using CO adsorption at low temperatures, a proven technique for characterizing solid acid catalysts.^67,68^Fig. 9 displays the difference infrared (IR) spectra of the SAO materials, obtained by subtracting the spectra of samples with adsorbed CO from the corresponding spectra of samples degassed at 673 K (The spectrum of the dehydrated AlPO STA-1 shows very little signal in the hydroxyl region, Fig. S10†). The SAPO and GeAPO samples contain acid sites, as shown by signals in the Brønsted hydroxyl range in the dehydrated sample and the negative intensities of these difference spectra appearing in the range 3690 and 3640 cm^−1^ when the acid sites interact with CO molecules, whereas there is no evidence of significant Brønsted acid interactions for the AlPO. This may be taken as strong evidence for the isomorphic substitution of Si and more remarkably Ge in framework positions. The red-shift of the Si(Ge)–OH is a good indication of the acid strength of the adsorption site, as this shift becomes greater for more acidic sites. From this, SAPO STA-1 is observed to exhibit stronger acid sites than GeAPO STA-1 (and GeAPO STA-1 than AlPO STA-1). This order of acid strength is further confirmed by the blue shift of the characteristic CO band at 2175 cm^−1^, which corresponds to the perturbed vibration of CO molecules upon adsorption on acid sites. As the acid strength of the AlPO-based STA-1 materials decreases, the IR band at approximately 2175 cm^−1^, assigned to adsorbed CO, appears at lower frequencies, which agrees with the order of increasing acidity (AlPO < GeAPO < SAPO).

**Fig. 10:**
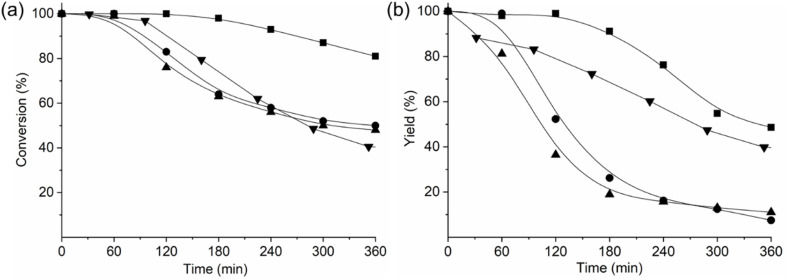
(a) Conversion of cyclohexanone oxime and (b) yield to ε-caprolactam (10 g L^−1^ solution of cyclohexanone oxime in ethanol flowing at a WHSV of 0.79 g_reactant_ g_cat_^−1^ h^−1^) at 598 K as a function of time on stream for the catalysts AlPO STA-1 (■), SAPO STA-1 (●), GeAPO STA-1 (▲) and SAPO-37 (▼) (from ref. 13).

To evaluate these AlPO-based materials further, we conducted catalytic tests for the Beckman rearrangement of cyclohexanone oxime in a plug-flow reactor. The activities and product yields of these catalysts were compared with the reported catalytic performance of a microporous SAPO-37 catalyst with a similar Si content, exhibiting weak to medium acid strength (Fig. 10).^13^ We found that AlPO STA-1 showed the highest activity and the least catalytic deactivation among the STA-1 catalysts, even outperforming that reported for the microporous form of SAPO-37 (although the mesoporous form of SAPO-37 displays much slower deactivation).^13^ The fast deactivation of SAPO STA-1 and GeAPO STA-1 can be attributed to the formation of polymerization residues that poison their acid sites. Indeed, the SAPO and GeAPO catalysts contained *ca.* 25 wt% of occluded organic material after reaction, as indicated by TGA. In addition, the selectivity over SAPO and GeAPO STA-1 catalysts diminishes as a consequence of formation of secondary products, such as cyclohexanone and 2-cyclohexen-1-one, 5-hexenenitrile and aniline, which were not observed in AlPO STA-1 and SAPO-37.

These results indicate that the presence of acid sites on SAPO STA-1 and GeAPO STA-1 catalysts has a negative effect on their catalytic performance due to secondary reactions that result in low selectivity and rapid catalyst deactivation. The AlPO STA-1 catalyst displayed a significantly slower catalytic deactivation and higher selectivity than SAPO and GeAPO STA-1 materials. These results can be attributed to the presence of the very weak acid sites in AlPO STA-1, which, although sufficiently strong for catalysing the reaction, preclude the formation of secondary products and reduce caprolactam polymerisation, resulting in a more competitive catalyst for this process.^69,70^ Indeed, when the AlPO STA-1 catalyst is compared to the microporous SAPO-37 reported in literature, a higher yield of the desired ε-caprolactam is obtained.

Finally, it is important to note the remarkable hydrolytic stability of AlPO STA-1 upon calcination, allowing it to tolerate prolonged exposure to atmospheric moisture (up to two weeks in this study) without experiencing any loss of crystallinity. Additionally, both AlPO STA-1 calcined *in situ* (and not exposed to atmospheric moisture) and AlPO STA-1 calcined and subsequently exposed to air, show identical catalytic activities in the Beckman rearrangement reaction. By contrast, a calcined sample of SAPO-37 that we prepared for comparison as a catalyst (ESI, Section S3, Fig. S11†) rapidly became amorphous upon exposure to moist air (Fig. S12†) in line with the low reported stability of SAPO-37.^35^ This resulted in a strong decline in its catalytic performance.

## Conclusions

An aluminophosphate with the SAO topology type (AlPO STA-1) has been prepared for the first time, using *N*,*N*′-diethylbicyclo[2.2.2]oct-7-ene-2,3:5,6-dipyrrolidine (DEBOP) as the organic structure directing agent and in the presence of fluoride. The extra-framework species can be removed by calcination to leave a fully tetrahedrally-coordinated microporous solid with a high pore volume (0.36 cm^3^ g^−1^). Additionally, the material retains crystallinity upon exposure to moist air, which is not the case for its metalloaluminophosphate analogues.

The high crystallinity and strict Al–P ordering makes AlPO STA-1 highly suitable for structural characterisation by multinuclear NMR, in particular using ^27^Al and ^31^P MAS NMR, MQ NMR and ^27^Al–^31^P MQ INEPT correlation experiments, supported by DFT modelling of NMR parameters and powder X-ray diffraction. It is possible to assign all signals from framework cations using this NMR-crystallography approach, confirming the experimentally-determined structure. For the as-prepared aluminophosphate-fluoride the characterisation is complicated by positional disorder of OSDA molecules and charge balancing fluorides and more detailed NMR analyses are ongoing to resolve the structure of this as-prepared material. Nevertheless, it is possible to confirm the framework structure is SAO by modelling the OSDA location and its effect on the PXRD pattern, and NMR indicates that the F^−^ ions coordinate to framework Al, giving 5-fold AlO_4_F coordinated species, where they are thought to occupy Al–F–Al bridging sites and distort the framework. Calcination removes these and allows the framework to adopt its energy minimum configuration.

Additional synthetic work indicates that a small amount of substitution of Si and Ge is possible on the P sites. The GeAPO STA-1 is thought to be the first reported 3D large pore AlPO with Ge in the framework. Low temperature IR experiments using the probe molecule CO indicate that, while there are only a few, very weakly acidic hydroxyl groups in the AlPO STA-1, the GeAPO and SAPO materials possess some mildly acidic sites. Frameworks with three-dimensionally connected 12-ring channel systems are relatively rare, so the aluminophosphate forms of STA-1 are potential catalysts for reactions that require only weak acidity. Here their performance in the Beckmann rearrangement of cyclohexanoneoxime has been measured. It is found that the SAPO and GeAPO variants are active for the conversion, but deactivate rapidly with time on stream, as observed for a SAPO-37 catalyst. By contrast, the AlPO deactivates much more slowly. In addition, the selectivity over SAPO and GeAPO STA-1 catalysts diminishes as a consequence of formation of secondary products, such as cyclohexanone and 2-cyclohexen-1-one, 5-hexenenitrile and aniline, which were not observed in AlPO STA-1. The improved performance of AlPO STA-1 is therefore attributed to the lack of active site reactions that give rise to secondary products, while possessing sufficient (very weak) acidity capable of catalysing the Beckmann reaction at the high temperature used.

In the light of these measurements, and previous reports, a hierarchical meso- and microporous AlPO STA-1 could be an excellent catalyst for the Beckmann rearrangement. Such a catalyst could combine low deactivation rate, high activity and selectivity with resistance toward exposure to atmospheric moisture in the calcined state.

## Conflicts of interest

There are no conflicts of interest to declare.

## Supplementary Material

TA-012-D4TA01132E-s001

TA-012-D4TA01132E-s002
