# Supplementary material for: Synthesis of the large pore aluminophosphate STA-1 and its application as a catalyst for the Beckmann rearrangement of cyclohexanone oxime
Source: J Mater Chem A Mater. 2024 May 13;12(25):15398–411. doi: 10.1039/d4ta01132e (PMC11197783; doi:10.1039/d4ta01132e)
Supplement: TA-012-D4TA01132E-s001 [file TA-012-D4TA01132E-s001.pdf]

Electronic Supplementary Material (ESI) for Journal of Materials Chemistry A.
This journal is © The Royal Society of Chemistry 2024

Supporting Information

Synthesis of the Large Pore Aluminophosphate STA-1 and its Application as a Catalyst for the Beckmann Rearrangement of Cyclohexanone Oxime

Nuria Gonzalez-Camuñas,[1,2] Angel Cantin,[1] Daniel M. Dawson,[3] Magdalena M. Lozinska,[3] Joaquin Martínez-Triguero,[1] James Mattock,[4] Paul A. Cox,[4] Sharon E. Ashbrook,[3] Paul A. Wright[3,*] and Fernando Rey[1,*]

[1] *Instituto de Tecnología Química (ITQ), Universitat Politècnica de València (UPV) – Consejo Superior de Investigaciones Científicas (CSIC), 46022 Valencia, Spain*

[2] *Instituto de Tecnología Cerámica, Universidad Jaume I, Campus Universitario Riu Sec, Avda. Vicente Sos Baynat s/n, 12006, Castellón, Spain*

[3] *EaStCHEM School of Chemistry, University of St Andrews, Purdie Building, St Andrews, KY16 9ST, United Kingdom*

[4] *School of Pharmacy and Biomedical Sciences, University of Portsmouth, Portsmouth, PO1 2DT, United Kingdom*

* email addresses: paw2@st-andrews.ac.uk; frey@itq.upv.es

S1. Computational Modelling of N,N'-diethylbicyclo[2.2.2]oct-7-ene-2,3:5,6-dipyrrolidine (DEBOP)

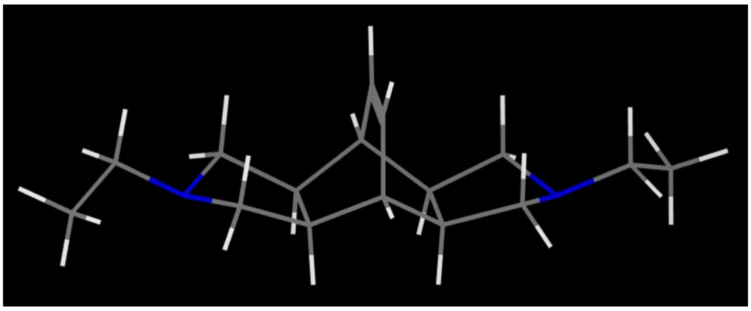

Figure S1. Modelled lowest energy configuration of the N,N'-diethylbicyclo[2.2.2]oct-7-ene-2,3:5,6-dipyrrolidine (DEBOP) OSDA, outside of the zeotype channel system.

Table S1. Calculated energies of 1 – 4 DEBOP OSDAs included per unit cell of $AlPO_4$ STA-1 indicate that the filling of all four positions within the unit cell provide favourable binding. All Energies in kcal /mol of OSDA.

Number of OSDAs	Total Energy of System	Total Free OSDA Energy	Total Binding Energy
1	–95.91	–56.90	–39.01
2	–191.24	–113.80	–77.44
3	–291.02	–170.70	–120.31
4	–392.91	–227.60	–165.31

S2. Characterisation of STA-1 (SAO Topology) Materials

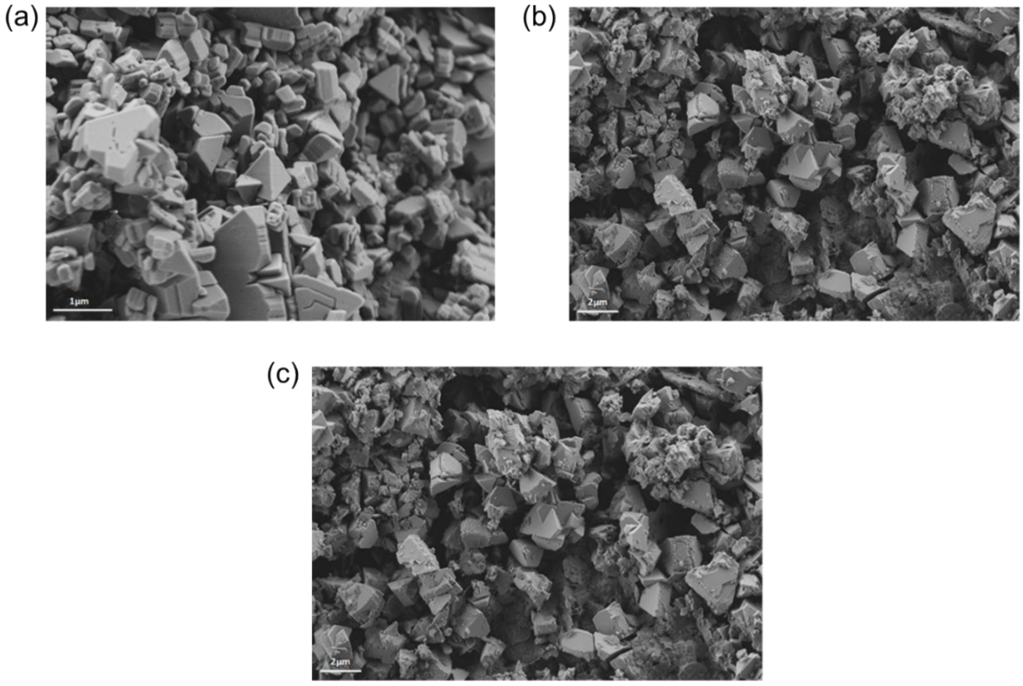

Figure S2. SEM images of (a) AlPO, (b) SAPO and (c) GeAPO STA-1 materials.

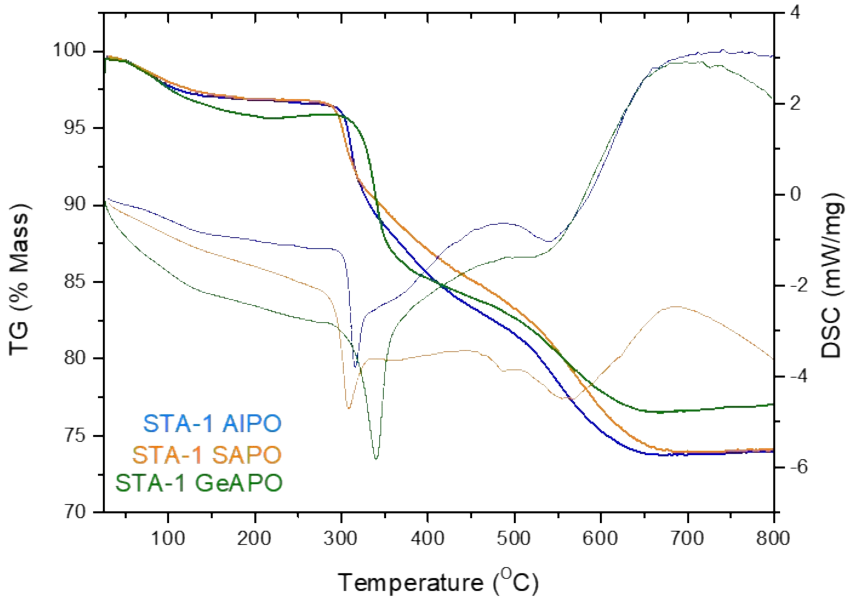

Figure S3. TG/DSC curves of AlPO, SAPO and GeAPO STA-1 materials.

Table S2. Chemical analysis of all STA-1 samples.

	AlPO STA-1	SAPO STA-1	GeAPO STA-1
Al (wt%)[a]	16.76	17.39	17.33
P (wt%)[a]	18.01	16.06	14.74
M (=Si, Ge) (wt%)[a]	-	1.08	3.44
C (wt%)[b]	16.85	16.68	15.06
N (wt%)[b]	2.46	2.51	2.18
H (wt%)[b]	2.81	2.79	2.76
Al (atomic %)[c]	10.51	-	-
P (atomic %)[c]	11.84	-	-
F (atomic %)[c]	1.91	-	-

[a] From ICP-AES
[b] From CHN analysis
[c] From SEM-EDS

Table S3. Crystallographic details of dehydrated samples.

	As made AlPO STA-1 (SAO)	Calcined AlPO STA-1 (SAO)
Unit cell	$Al_{28}P_{28}O_{112}C_{77}N_{9.6}F_{0.3}$	$Al_{28}P_{28}O_{112}$
Temperature/K	298	298
Space group	*P* -4 *n* 2	*P* -4 *n* 2
X-ray source	Cu K_{α_1}	Cu K_{α_1}
Diffractometer	Stoe STADI P	Stoe STADI P
Wavelength (Å)	1.54056	1.54056
a = b / Å	13.3148(9)	13.74317(10)
c / Å	22.0655(20)	21.8131(5)
Volume/Å^3	3911.9(7)	4119.94(16)
Rp	0.0728	0.0407
Rwp	0.0987	0.0549
χ^2	7.181	4.783

Table S4. Al-O and P-O bond lengths and O-Al-O and O-P-O bond angles for refined structure of calcined and dehydrated AlPO STA-1 (SAO).

Al-O and P-O / Å		O-Al-O and O-P-O (°)	
Al1_O1	1.685(15)	O1_Al1_O5	109.1(12)
Al1_O5	1.688(15)	O1_Al1_O6	109.1(11)
Al1_O6	1.722(16)	O1_Al1_O7	110.8(11)
Al1_O7	1.683(16)	O5_Al1_O6	110.5(11)
Al2_O9	1.740(14)	O5_Al1_O7	110.5(11)
Al2_O11	1.733(15)	O6_Al1_O7	108.9(11)
Al2_O12	1.692(15)	O9_Al2_O11	107.1(11)
Al2_O13	1.710(15)	O9_Al2_O12	107.9(10)
Al3_O3	1.705(15)	O9_Al2_O13	111.3(11)
Al3_O4	1.712(15)	O11_Al2_O12	109.1(11)
Al3_O10	1.716(16)	O11_Al2_O13	111.4(11)
Al3_O14	1.686(15)	O12_Al2_O13	109.9(11)
Al4_O2	1.700(10)	O3_Al3_O4	108.9(11)
Al4_O2	1.700(10)	O3_Al3_O10	107.8(12)
Al4_O8	1.703(11)	O3_Al3_O14	110.8(11)
Al4_O8	1.703(11)	O4_Al3_O10	110.7(11)
P1_O1	1.529(15)	O4_Al3_O14	109.7(11)
P1_O2	1.538(14)	O10_Al3_O14	109.0(11)
P1_O3	1.552(14)	O2_Al4_O2	112.4(13)
P1_O4	1.564(14)	O2_Al4_O8	108.6(7)
P2_O6	1.533(14)	O2_Al4_O8	107.3(7)
P2_O8	1.563(14)	O2_Al4_O8	107.3(7)
P2_O9	1.564(13)	O2_Al4_O8	108.6(7)
P2_O12	1.534(14)	O8_Al4_O8	112.7(13)
P3_O5	1.546(14)	O1_P1_O2	112.7(13)
P3_O7	1.505(15)	O1_P1_O3	110.1(12)
P3_O10	1.505(14)	O1_P1_O4	108.0(12)
P3_O11	1.563(15)	O2_P1_O3	107.6(12)
P4_O13	1.546(10)	O2_P1_O4	110.0(12)
P4_O13	1.546(10)	O3_P1_O4	108.4(12)
P4_O14	1.535(10)	O6_P2_O8	110.2(12)
P4_O14	1.535(10)	O6_P2_O9	108.8(12)
		O6_P2_O12	110.7(12)
		O8_P2_O9	110.6(12)
		O8_P2_O12	107.6(11)
		O9_P2_O12	108.9(11)
		O5_P3_O7	106.7(13)
		O5_P3_O10	110.8(13)
		O5_P3_O11	107.4(12)
		O7_P3_O10	107.1(13)
		O7_P3_O11	114.8(13)
		O10_P3_O11	110.0(11)
		O13_P4_O13	112.7(14)
		O13_P4_O14	109.9(8)
		O13_P4_O14	107.2(8)
		O13_P4_O14	107.2(8)

Table S5. Fractional atomic coordinates, occupancies, multiplicities and isotropic displacement parameters (in Å^2) for calcined and dehydrated AlPO STA-1 (SAO).

	Type	x	y	z	Occup.	Mult.	Uiso
P1	P	0.3890(12)	0.1167(12)	0.1330(7)	1.0	8	0.0077(4)
P2	P	0.1105(11)	0.1179(11)	0.1888(6)	1.0	8	0.0077(4)
P3	P	0.2037(11)	−0.1124(12)	0.0574(7)	1.0	8	0.0077(4)
P4	P	0.2957(10)	−0.2043(10)	0.25	1.0	4	0.0077(4)
Al1	Al	0.2039(12)	0.1145(13)	0.0591(8)	1.0	8	0.0077(4)
Al2	Al	0.1121(13)	−0.1150(12)	0.1883(7)	1.0	8	0.0077(4)
Al3	Al	0.3888(13)	−0.1110(13)	0.1350(7)	1.0	8	0.0077(4)
Al4	Al	0.2951(11)	0.2049(11)	0.25	1.0	4	0.0077(4)
O1	O	0.3198(10)	0.1375(15)	0.0798(7)	1.0	8	0.0077(4)
O2	O	0.3687(15)	0.1812(12)	0.1892(5)	1.0	8	0.0077(4)
O3	O	0.3792(10)	0.0091(10)	0.1536(6)	1.0	8	0.0077(4)
O4	O	0.4954(10)	0.1336(11)	0.1098(6)	1.0	8	0.0077(4)
O5	O	0.1937(10)	−0.0040(10)	0.0397(6)	1.0	8	0.0077(4)
O6	O	0.1285(14)	0.1367(14)	0.1205(6)	1.0	8	0.0077(4)
O7	O	0.1702(10)	0.1861(11)	0.0002(7)	1.0	8	0.0077(4)
O8	O	0.1777(11)	0.1846(12)	0.2286(9)	1.0	8	0.0077(4)
O9	O	0.1309(11)	0.0081(10)	0.2028(6)	1.0	8	0.0077(4)
O10	O	0.3056(10)	−0.1348(15)	0.0783(7)	1.0	8	0.0077(4)
O11	O	0.1301(13)	−0.1328(15)	0.1105(6)	1.0	8	0.0077(4)
O12	O	0.0048(10)	0.1413(10)	0.2059(6)	1.0	8	0.0077(4)
O13	O	0.1889(11)	−0.1858(12)	0.2309(9)	1.0	8	0.0077(4)
O14	O	0.3638(14)	−0.1819(12)	0.1961(5)	1.0	8	0.0077(4)

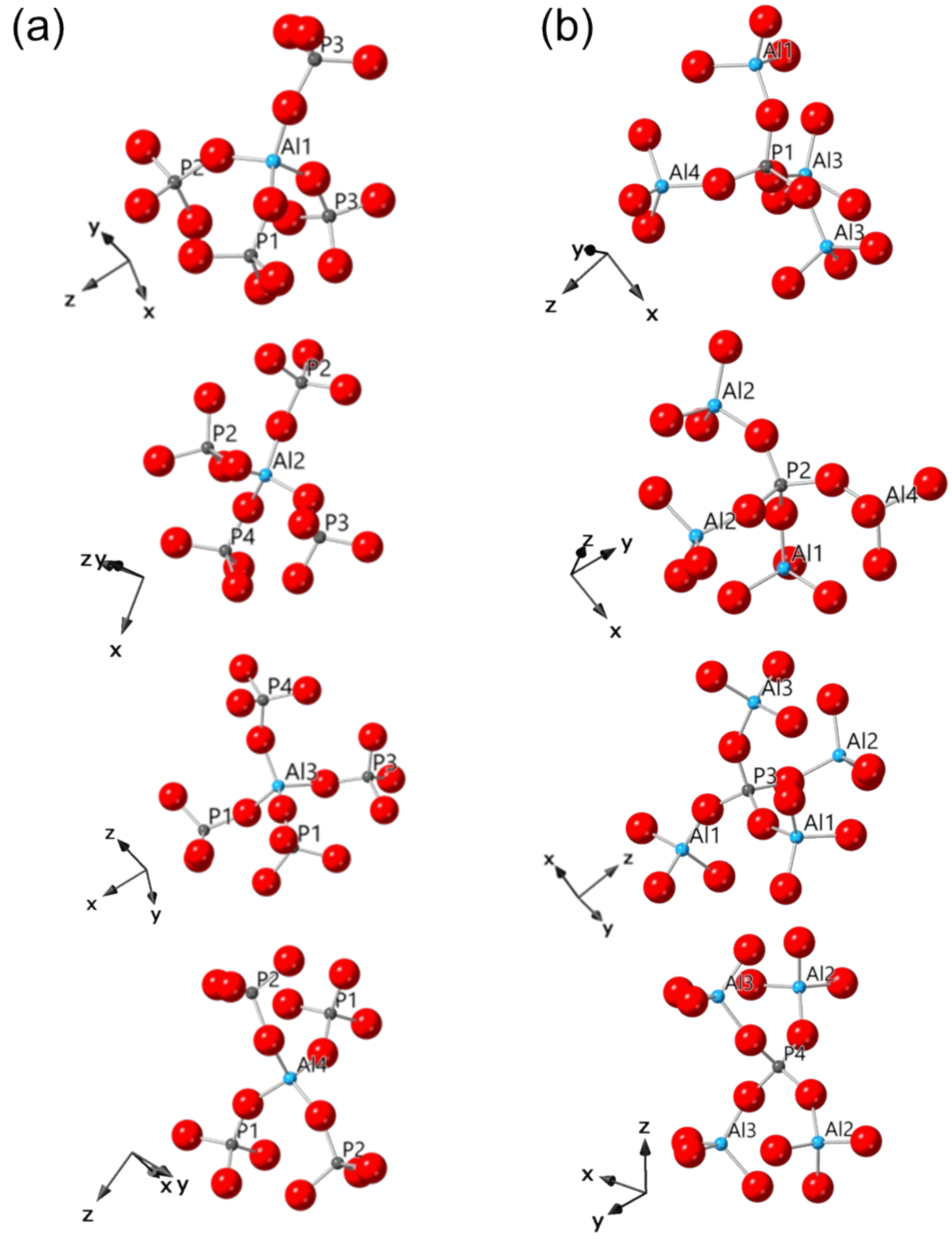

Figure S4. T-site nearest neighbour environments in $AlPO_4$ STA-1for (a) Al atoms and (b) P atoms. (Al = blue spheres, P = grey spheres and O = red spheres)

Figure S5. Fluoride ion located in the cage of the aluminophosphate fluoride UiO-7, which gives a ^{19}F MAS NMR signal at −109 ppm.[1,2]

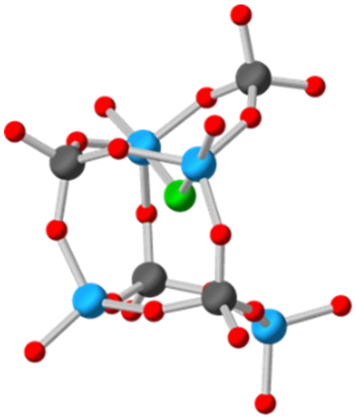

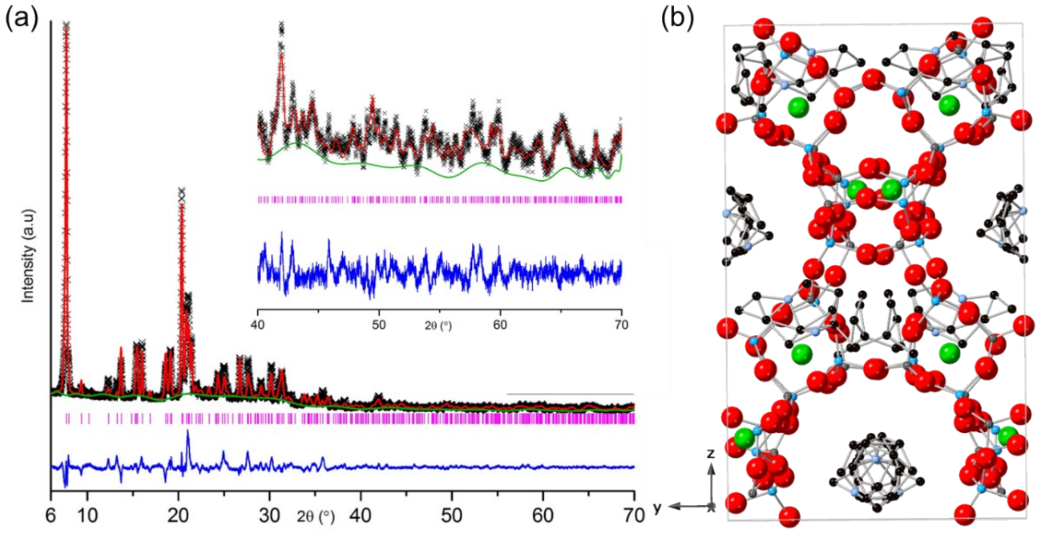

Figure S6. Rietveld plot of PXRD data (λ = 1.54056 Å, T = 298 K) of as-made and dehydrated AlPO STA-1 (SAO) (Observed – black, calculated – red, difference – blue, phase – pink and background – green). (b) Structure of as-made and dehydrated (*P*–4*n*2 symmetry) AlPO STA-1 (SAO) obtained from Rietveld refinement (Al = blue spheres, P = grey spheres, O = red spheres, C = black spheres, N = blue spheres and F = green spheres).

Table S6. Al-O and P-O bond lengths and O-Al-O and O-P-O bond angles for refined structure of as-made and dehydrated AlPO STA-1 (SAO).

Al-O and P-O / Å		O-Al-O and O-P-O (°)	
Al1_O1	1.67555(10)	O1_Al1_O5	105.189(1)
Al1_O5	1.72880(10)	O1_Al1_O6	106.231(5)
Al1_O6	1.73167(11)	O1_Al1_O7	127.073(1)
Al1_O7	1.66261(9)	O5_Al1_O6	102.817(2)
Al2_O9	1.70192(10)	O5_Al1_O7	107.445(4)
Al2_O11	1.68039(15)	O6_Al1_O7	105.689(5)
Al2_O12	1.72438(11)	O9_Al2_O11	111.103(2)
Al2_O13	1.72648(9)	O9_Al2_O12	108.373(1)
Al3_O3	1.67229(11)	O9_Al2_O13	110.133(4)
Al3_O4	1.69841(10)	O11_Al2_O12	107.592(1)
Al3_O10	1.79777(10)	O11_Al2_O13	119.055(3)
Al3_O14	1.74307(10)	O12_Al2_O13	99.514(2)
Al4_O2	1.69747(10)	O3_Al3_O4	129.121(0)
Al4_O2	1.69747(10)	O3_Al3_O10	103.213(0)
Al4_O8	1.68314(10)	O3_Al3_O14	118.639(3)
Al4_O8	1.68314(10)	O4_Al3_O10	100.893(4)
P1_O1	1.56632(9)	O4_Al3_O14	98.117(3)
P1_O2	1.47179(10)	O10_Al3_O14	103.096(5)
P1_O3	1.72738(11)	O2_Al4_O2	110.703(5)
P1_O4	1.50347(9)	O2_Al4_O8	111.792(5)
P2_O6	1.53726(13)	O2_Al4_O8	106.345(2)
P2_O8	1.52553(8)	O2_Al4_O8	106.345(2)
P2_O9	1.53151(9)	O2_Al4_O8	111.792(5)
P2_O12	1.51913(10)	O8_Al4_O8	109.944(1)
P3_O5	1.52901(9)	O1_P1_O2	109.413(5)
P3_O7	1.51110(11)	O1_P1_O3	114.475(0)
P3_O10	1.50017(8)	O1_P1_O4	109.951(5)
P3_O11	1.55385(9)	O2_P1_O3	108.589(3)
P4_O13	1.49668(8)	O2_P1_O4	119.189(2)
P4_O13	1.49668(8)	O3_P1_O4	94.745(1)
P4_O14	1.57515(9)	O6_P2_O8	109.061(4)
P4_O14	1.57515(9)	O6_P2_O9	108.627(1)
		O6_P2_O12	103.405(0)
		O8_P2_O9	113.158(3)
		O8_P2_O12	107.538(1)
		O9_P2_O12	114.519(1)
		O5_P3_O7	101.491(4)
		O5_P3_O10	112.519(1)
		O5_P3_O11	110.358(1)
		O7_P3_O10	107.540(3)
		O7_P3_O11	120.454(4)
		O10_P3_O11	104.670(5)
		O13_P4_O13	119.808(1)
		O13_P4_O14	102.301(5)
		O13_P4_O14	114.578(2)
		O13_P4_O14	114.578(2)
		O13_P4_O14	102.301(5)
		O14_P4_O14	102.319(5)

Table S7. Fractional atomic coordinates, occupancies, multiplicities and isotropic displacement parameters (in Å^2) for as-made and dehydrated AlPO STA-1 (SAO).

	Type	x	y	z	Occup.	Mult.	Uiso
P1	P	0.39211	0.15616	0.13658	1.0	8	0.00824
P2	P	0.10433	0.12339	0.18981	1.0	8	0.00824
P3	P	0.21688	−0.08312	0.05404	1.0	8	0.00824
P4	P	0.29651	−0.20349	0.25	1.0	4	0.00824
Al1	Al	0.20218	0.14954	0.05977	1.0	8	0.00824
Al2	Al	0.12421	−0.10084	0.17968	1.0	8	0.00824
Al3	Al	0.4075	−0.09623	0.1383	1.0	8	0.00824
Al4	Al	0.28042	0.21958	0.25	1.0	4	0.00824
O1	O	0.31263	0.18785	0.08786	1.0	8	0.00824
O2	O	0.36784	0.20449	0.19475	1.0	8	0.00824
O3	O	0.40128	0.02816	0.14807	1.0	8	0.00824
O4	O	0.49629	0.1668	0.11108	1.0	8	0.00824
O5	O	0.22243	0.02756	0.03587	1.0	8	0.00824
O6	O	0.12312	0.13398	0.12137	1.0	8	0.00824
O7	O	0.13357	0.2064	0.00698	1.0	8	0.00824
O8	O	0.16298	0.20474	0.22324	1.0	8	0.00824
O9	O	0.13329	0.01685	0.20927	1.0	8	0.00824
O10	O	0.3126	−0.12002	0.08215	1.0	8	0.00824
O11	O	0.1359	−0.09873	0.10386	1.0	8	0.00824
O12	O	−0.00664	0.14761	0.19633	1.0	8	0.00824
O13	O	0.19681	−0.18351	0.22108	1.0	8	0.00824
O14	O	0.37218	−0.17424	0.19805	1.0	8	0.00824
C1	C	1.03265	0.62325	0.45801	0.60148	8	0.09124
C2	C	0.95265	0.54294	0.45512	0.60148	8	0.09124
C3	C	1.00426	0.44768	0.43449	0.60148	8	0.09124
C4	C	1.0565	0.46316	0.37115	0.60148	8	0.09124
C5	C	1.03621	0.56789	0.34679	0.60148	8	0.09124
C6	C	1.0807	0.63954	0.39355	0.60148	8	0.09124
C7	C	1.06749	0.75248	0.38342	0.60148	8	0.09124
C8	C	1.00164	0.72964	0.47677	0.60148	8	0.09124
C9	C	1.01786	0.374	0.33338	0.60148	8	0.09124
C10	C	0.93847	0.35527	0.42313	0.60148	8	0.09124
C11	C	0.94935	0.20938	0.35464	0.60148	8	0.09124
C12	C	0.92327	0.13345	0.40421	0.60148	8	0.09124
C13	C	1.05985	0.89847	0.4525	0.60148	8	0.09124
C14	C	1.14536	0.95749	0.42388	0.60148	8	0.09124
C15	C	0.92304	0.58367	0.34846	0.60148	8	0.09124
C16	C	0.88082	0.57163	0.40354	0.60148	8	0.09124
N1	N	1.07356	0.79194	0.44523	0.60148	8	0.09124
N2	N	0.99598	0.29844	0.37902	0.60148	8	0.09124
F3	F	0.24767	−0.0578	0.16537	0.03997	8	0.05

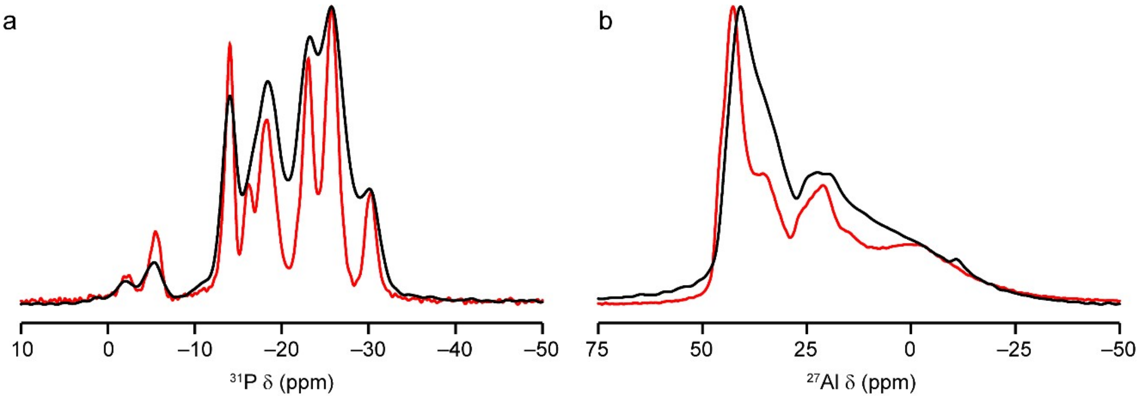

Figure S7. (a) ^{31}P (9.4 T, 14 kHz MAS) and (b) ^{27}Al (14.1 T, 14 kHz MAS) NMR spectra of (black) SAPO STA-1 and (red) AlPO-STA-1.

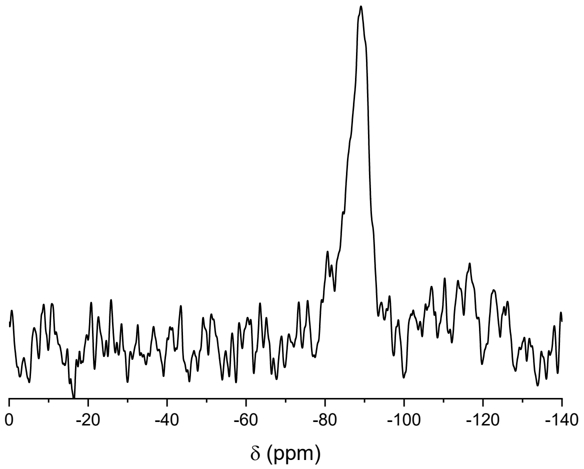

Figure S8. ^{29}Si (9.4 T, 14 kHz MAS) NMR spectrum of SAPO STA-1.

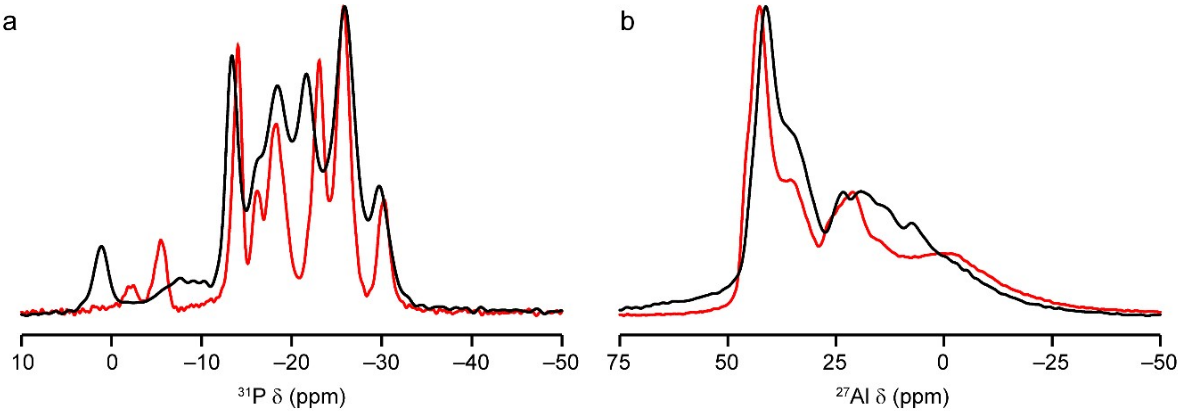

Figure S9. (a) ^{31}P (9.4 T, 14 kHz MAS) and (b) ^{27}Al (14.1 T, 14 kHz MAS) NMR spectra of (black) GeAPO STA-1 and (red) AlPO-STA-1.

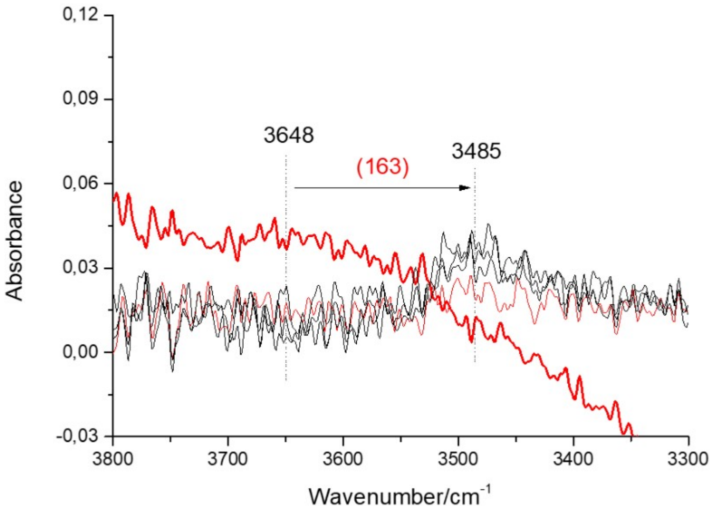

Figure S10. IR spectrum of dehydrated AlPO STA-1 in the hydroxyl region (red, bold) and difference spectra after low temperature adsorption of CO at different pressures.

S3. Comparative studies with SAPO-37

For the synthesis of SAPO-37, 0.2571 g of water, 0.6918 g phosphoric acid (85 wt %) and 0.1802 g of fumed silica were mixed at RT for 30 mins. Then 0.6118 g of alumina (76.5 wt %) was added very slowly with vigorous stirring. The mixture was stirred for further 4 hours at RT and then 3.0506 g tetrapropylammonium hydroxide (40% solution) and 0.0547 g tetra-methylammonium hydroxide were added. The final gel pH was approximately 7. The mixture was then stirred for further 2 hours and the gel was sealed in a Teflon-lined autoclave and heated at 463 K for 48 h. The overall gel composition was 2 H_3PO_4 : SiO_2 : 2 $Al(OH)_3$: 0.05 TMAOH: 2 TPAOH: 50 H_2O. The resulting solid was isolated by centrifugation, washed with distilled water and dried at 333 K overnight. In order to remove the OSDA, the solid was calcined at 923 K for 12 h in air. ICP-AES indicated an inorganic composition of $Al_{1.02}Si_{0.34}P_{0.60}O_4$.

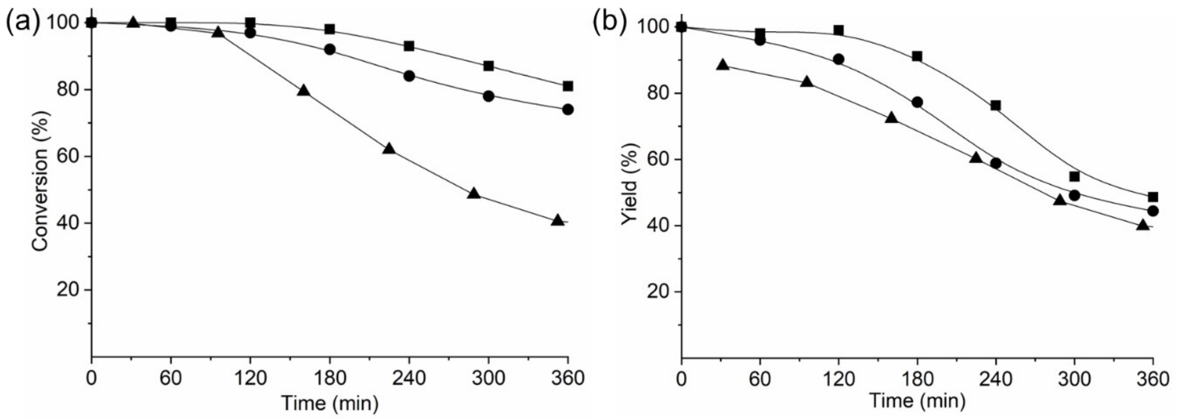

Figure S11. Comparison of catalytic activity of AlPO-SAO and SAPO-37 with different Si content, at 598 K: AlPO STA-1 (■), SAPO-37 (●; high Si content) and SAPO-37 (▲; low Si content; from Angew. Chem. Int. Ed. 2020, 59, 19561–19569). Conversion of cyclohexanone oxime and yield to ε-caprolactam (10 g/L solution of cyclohexanone oxime in ethanol at a WHSV of 0.79 $g_{reactant}\cdot g_{cat}^{-1}\cdot h^{-1}$) at 598 K as a function of time on stream.

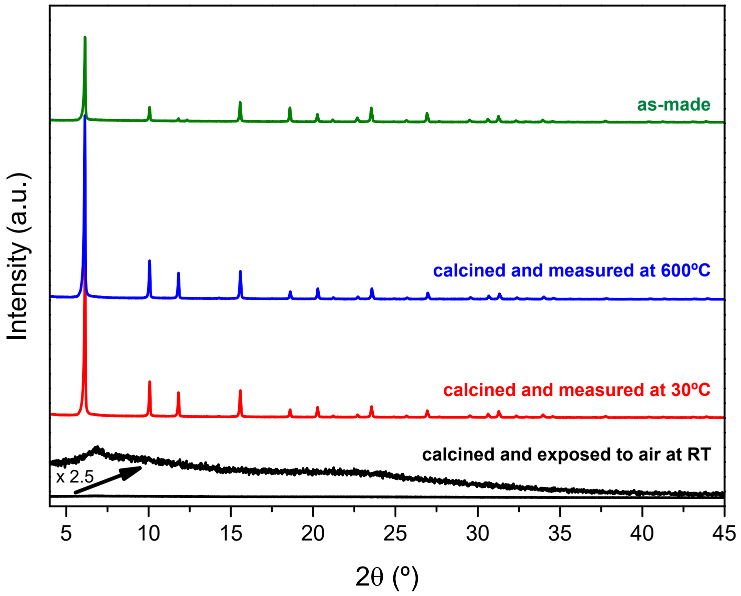

Figure S12. PXRD patterns of the SAPO-37 sample collected using an Anton-Paar XRK-900 reaction chamber attached to a Malvern-Panalytical Empyrean diffractometer with a PIXcel detector. Calcination was performed under a constant flow of a dried 1:4 v/v O_2/N_2 stream.

References

[1] D. E. Akporiaye, H. Fjellvag, E. N. Halvorsen, J. Hustveit, A. Karlsson and K. P. Lillerud, UiO-7: A New Aluminophosphate Phase Solved by Simulated Annealing and High Resolution Powder Diffraction, *J. Phys. Chem.* 1996, **100**, 16641–16646

[2] A. Bailly, J. P. Amoureux, J. W. Wiench and M. Pruski, Structural Analysis of ZON-Type Aluminophosphates by Solid State NMR, *J. Phys. Chem. B*, 2001, **105**, 773–776.
